# A Review of Computational Approaches to the Microstructure-Informed Mechanical Modelling of Metals Produced by Powder Bed Fusion Additive Manufacturing

**DOI:** 10.3390/ma16196459

**Published:** 2023-09-28

**Authors:** Olga Zinovieva, Varvara Romanova, Ekaterina Dymnich, Aleksandr Zinoviev, Ruslan Balokhonov

**Affiliations:** 1 School of Engineering and Technology, University of New South Wales Canberra, Campbell, ACT 2612, Australia; a.zinoviev@adfa.edu.au; 2 Institute of Strength Physics and Materials Science, 634055 Tomsk, Russia; dymnich@ispms.ru (E.D.); rusy@ispms.ru (R.B.)

**Keywords:** metal additive manufacturing, powder bed fusion, microstructure modelling, micromechanics, crystal plasticity, microstructure-based simulation

## Abstract

In the rapidly evolving field of additive manufacturing (AM), the predictability of part properties is still challenging due to the inherent multiphysics complexity of the technology. This results in time-consuming and costly experimental guess-and-check approaches for manufacturing each individual design. Through synthesising advancements in the field, this review argues that numerical modelling is instrumental in mitigating these challenges by working in tandem with experimental studies. Unique hierarchical microstructures induced by extreme AM process conditions– including melt pool patterns, grains, cellular–dendritic substructures, and precipitates—affect the final part properties. Therefore, the development of microstructure-informed mechanical models becomes vital. Our review of numerical studies explores various modelling approaches that consider the microstructural features explicitly and offers insights into multiscale stress–strain analysis across diverse materials fabricated by powder bed fusion AM. The literature indicates a growing consensus on the key role of multiscale integrated process–structure–property–performance (PSPP) modelling in capturing the complexity of AM-produced materials. Current models, though increasingly sophisticated, still tend to relate only two elements of the PSPP chain while often focusing on a single scale. This emphasises the need for integrated PSPP approaches validated by a solid experimental base. The PSPP paradigm for AM, while promising as a concept, is still in its infantry, confronting multifaceted challenges that require in-depth, multidisciplinary expertise. These challenges range from accounting for multiphysics phenomena (e.g., advanced laser–material interaction) and their interplay (thermo-mechanical and microstructural evolution for simulating Type II residual stresses), accurately defined assumptions (e.g., flat molten surface during AM or purely epitaxial solidification), and correctly estimated boundary conditions for each element of the PSPP chain up to the need to balance the model’s complexity and detalisation in terms of both multiphysics and discretisation with efficient multitrack and multilayer simulations. Efforts in bridging these gaps would not only improve predictability but also expedite the development and certification of new AM materials.

## 1. Introduction

Powder bed fusion (PBF), conceived as the most mature metal additive manufacturing (MAM) technology [1], is actively paving its way in aerospace, automotive, health, defence, and other industries by offering unique opportunities not seen in conventional formative and subtractive manufacturing [2,3,4]. PBF manufactures complex-shaped metal parts layer-by-layer, melting thin layers of powder, which correspond to slices of computer-aided design (CAD) models, using either a laser or electron beam—thus subdividing PBF into laser powder bed fusion (LPBF) and electron beam powder bed fusion (EPBF). Among PBF’s benefits are the production of easily customisable intricate metal parts, on-demand manufacturing at the point of need, shortened cycle times, efficient material usage, and advanced digital manufacturing capabilities. Furthermore, PBF breaks new ground to engineer microstructures and tailor properties to meet specific performance requirements, facilitating the development of innovative components [5,6].

However, the benefits of PBF technology come at a price. During manufacturing, PBF induces multiple interconnected multi-physics phenomena to co-occur simultaneously, locally, and within very short timescales, including melting and re-melting, the evaporation and emission of a material, directional solidification, chemical reactions, thermo-capillarity, surface tension, and many others [7,8]. The complex physics of PBF which is closely linked with numerous process parameters [9,10] affects the final product’s microstructure and properties and makes the PBF process challenging to control. Even small changes in parameters might trigger unexpected and previously unexplored effects and unwanted defects in a resulting component, making predictability a significant challenge. This is often inappropriate for high-demanding sectors such as the aerospace and health industries [11,12]. MAM still suffers from many scientific, technological, and economic issues, which underscores the need for a sophisticated understanding of the technology’s physics [13].

PBF AM produces a material from a raw powder together with a component. This yields a unique hierarchical microstructure of a near-net-shaped final part which is often characterised by a pronounced morphological and crystallographic texture [4,5,6,14,15,16,17,18,19]. Being a complex unknown function of multiple process parameters, this microstructure dramatically complicates the prediction of mechanical properties of AM parts. Given the numerous multi-physics processes involved, there is a pressing need to shift from experimental guesswork for each individual design to a more predictable approach. The complexity of accurate predictions is further pronounced by the inherently multiscale physics of the AM process, from the formation of nanoscale precipitations impacting the mechanical behaviour up to kilometres of heat source path and thousands of layers in a single part [2]. Seminal works [2,4,13,20,21,22,23] emphasise the need for the prediction of the whole processing–(micro)structure–property–performance (PSPP) chain, including the development and application of sophisticated numerical models for this purpose. ‘Extraordinary value of AM computational models’ stems from the opportunity of the process digital reconstruction to overcome the challenges of experimental research, including its high cost, complexity, and the difficulties related to in situ process monitoring (internal temperature distribution, stress state, etc.) [23]. However, numerical models developed for traditional materials and manufacturing processes require adaptations for the new class of materials and the additive way of manufacturing.

The mechanics of materials traditionally divides multiscale approaches for modelling the material’s behaviour into two groups. Homogenisation approaches estimate the macroscopic stress–strain relationship in a material taking its internal structure into account indirectly. They do it either through mathematical terms in constitutive equations (analytical and semi-analytical homogenisation) [17,24,25,26,27,28] or through solving the boundary value problems (BVPs) for a representative volume element (RVE) using the methods of computational homogenisation [25,29,30,31,32]. The key advantage of homogenisation methods lies in their applicability to large components, which makes them interesting for industrial applications. However, these models do not allow us to capture local stresses and strains that can significantly deviate from the homogenised response.

Local stress–strain fields can be analysed using the second group of methods which directly account for microstructural features. This type of computational analysis is often termed microstructure-based (microstructure-informed) mechanical modelling [33,34,35] or micromechanical simulation [29]. It provides valuable information on spatial stress–strain distributions and potential crack initiation sites [29,32]. The mechanical performance of AM parts is influenced by a combination of microstructural details, including grain size, orientation and spatial arrangement, substructure (dendrites, cells, precipitates, etc.), and porosity [34]. Recent studies have highlighted the vital role of considering the realistic microstructure to investigate deformation and fracture in AM materials [32,36]. Notably, microstructure-informed models can also be applied to predict the macroscopic behaviour of a material, including the effective mechanical properties, stress–strain curves, and yield locus with computational homogenisation methods [31,36].

In light of the above, this contribution details methods of microstructure-informed mechanical simulations applied to PBF-produced materials. Similar to the seminal review [37], this contribution addresses a continuous description of AM materials, omitting the atomistic scale from consideration. To the best of our knowledge, no standalone review exists on micromechanical simulations of AM materials, although general reviews on simulations of MAM have touched on computational homogenisation. For example, the review [38] considers the prediction of mechanical properties of AM materials, including a precipitate evolution-based model applied to solid-state friction stir AM [39], a dislocation density-evolution based model employed to describe residual stresses induced during the direct energy deposition (DED) of Ti-6Al-4V alloy without explicit consideration of its microstructure [28], and a crystal plasticity (CP) model but does not discuss any applications of the CP model to AM. A section of [40] summarises modelling efforts for monotonic and cyclic loading conditions without considering the models in detail. In their recent review [41], Zhao and coworkers summarised computational efforts in microstructure-based simulations of an LPBF AlSi10Mg alloy.

The review provides an experimental background on PBF for various alloys (Section 2), an exploration of the PSPP concept (Section 3), a summary of the approaches involved in the generation of AM model microstructures (Section 4), an overview of micromechanical modelling approaches (Section 5), and their insights on the deformation behaviour of PBF-produced materials (Section 6). Section 7 summarises the review and outlines future directions for the community to address.

## 2. Microstructural Features of Materials Fabricated by PBF

**Figure 1:**
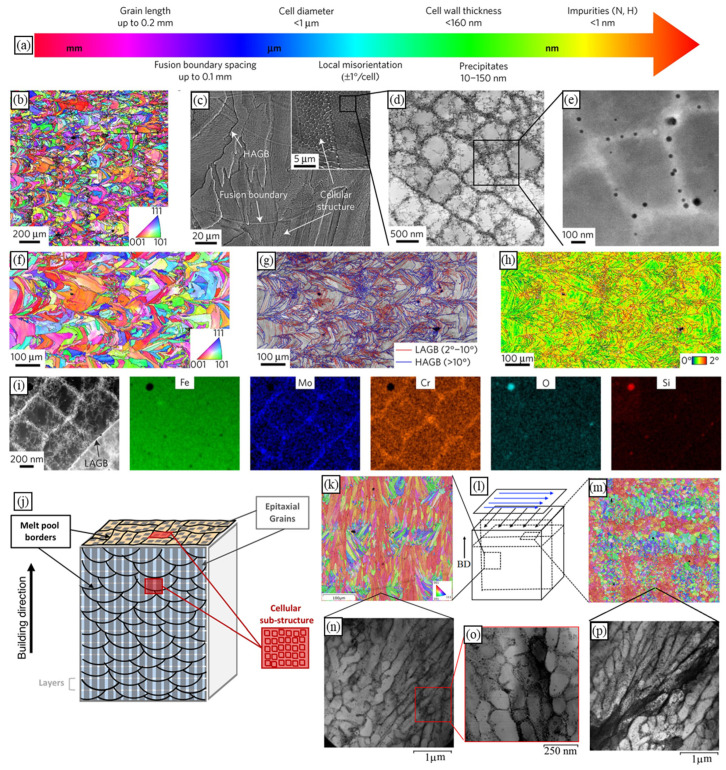
Illustration of typical hierarchical microstructures in additively manufactured metal parts. Images (**b**–**i**) describe LPBF stainless steel [18] and (**k**–**p**) describe LPBF AlSi10Mg over (**a**) a variety of length scales. Features include: (**b**,**f**,**k**,**m**) cross-sectional electron backscatter diffraction (EBSD) orientation maps showing grain orientations; (**c**) a scanning electron microscopy (SEM) image showcasing fusion boundaries, high-angle grain boundaries (HAGBs), and cellular–dendritic structures; (**d**) a bright-field transmission electron microscopy (TEM) image of cells; (**e**) a high-angle annular dark-field (HAADF) scanning TEM (STEM) image of the cells demonstrated in (**d**); (**g**) an EBSD image quality map showing HAGBs and low-angle grain boundaries (LAGBs); (**h**) a map of the kernel average misorientation showing the local misorientation across a grain; (**i**) an HAADF STEM image showing the segregation of individual elements; (**j**) schematics of the hierarchy of LPBF microstructures [44]; (**l**) a schematics describing the location of different cross-sections in an AlSi10Mg sample along with the scanning strategy used for manufacturing; and (**n**–**p**) microstructural images. The images are taken in sections (**b**–**h**,**k**,**n**,**o**) parallel and (**m**,**p**) perpendicular to the build direction (BD), where (**c**–**e**,**k**,**n**,**o**) the BD is vertical and (**b**,**f**–**h**) horizontal.

PBF induces unique thermal behaviour, including high cooling and heating rates, repeated melting, and large temperature gradients. This fosters complex hierarchical microstructures that often result in anisotropic mechanical properties [42]. The microstructure hierarchy might span up to six orders of magnitude, from a melt pool pattern, grains, and pores (up to mm) to cellular–dendritic sub-grain structures (µm) and precipitates (nm) [18,43,44] (Figure 1). Note that this section aims to summarise key microstructural features that affect the mechanical behaviour of PBF-produced materials and, therefore, should be considered in numerical simulations. Detailed discussions are available in several reviews [3,19,41,42,43,45,46,47].

### 2.1. Melt Pool Pattern

Generally, PBF manufactures parts by locally melting raw material layer-by-layer to generate the height and hatch-by-hatch to produce the layer. A melt pool pattern (Figure 1c,j) evident in many as-built alloy systems under optical microscopy impacts lower scales of the microstructure and the mechanical response of AM materials due to local process-induced thermal conditions. A melt pool can be split into the bulk of the melt pool and the boundary region [48]. In addition, the heat-affected zone (HAZ) is often considered separately [43]. Differences between the microstructures near the melt pool boundary and in the bulk are significant. The reason might be the presence of the remelted and reheated regions of the previous layer generating locally varying thermal conditions. For instance, some studies suggested that the low mobility of the solid–liquid interface at the melt pool boundary led to a planar solidification zone in the boundary region for PBF-produced Al-Cu-Mg [49], Ni-Nb [50], Ti-6Al-4V, and Ti-6Al-4V + 10Mo alloys [51] and 316L steel [52]. However, planar morphology features were not detected in laser surface engineered IN718 superalloy despite the fulfilment of the planar solidification conditions (the high value of the temperature gradient to solidification rate ratio G/R≥ 7000 K × s × mm^–2^ suggested in [53]) [54].

The bulk of LPBF-fabricated AlSi10Mg is characterised by a fine substructure, and melt pool boundaries are characterised by a coarse substructure, with the HAZ containing coarse Si particles [19,41,48]. Melt pool boundary regions characterised by high cooling rates and large temperature gradients exhibit a non-uniform solute distribution. For example, Thijs et al. [55] observed the segregation of Al at melt pool boundaries of LPBF-produced Ti-6Al-4V that resulted in Ti_3_Al intermetallic phase precipitation. Some AM aluminium alloys are known for their inhomogeneous grain pattern with the distinct difference between the bulk and boundary of the melt pool (Figure 1k). This difference might be caused by an inoculant compound with a high melting temperature (e.g., Si in Al-Si alloys), which first precipitates in the melt or remains undissolved close to the fusion boundary during solidification [56,57]. This phase acts as a site for heterogeneous nucleation near the melt pool boundary. Some researchers explain the presence of fine equiaxed grains at the fusion boundary by purely thermal conditions drawing a parallel with a chill zone [58], while others disagree, supporting their position with the zero cooling rate at the melt pool boundary [57].

Some studies consider melt pools as separate microstructural elements, showing that they tend to shift relative to each other under load [30]. A number of studies are devoted to the role of melt pool boundaries in the mechanical behaviour of AM materials (see, e.g., [59,60,61]). Cracks often form and propagate along melt pool boundaries both during the printing and under loading of as-built samples [48,59,60,61,62,63]. Xiong et al. [61] concluded that not the texture but the distribution of melt pool boundaries on a load-bearing face predominantly affects the anisotropy of mechanical properties of an LPBF Al-Si alloy. Shifeng et al. [60] suggested that melt pool boundaries made a pronounced contribution to plastic deformation in as-built components due to a weaker bonding force between them as compared to grain boundaries.

### 2.2. Grains

**Figure 2:**
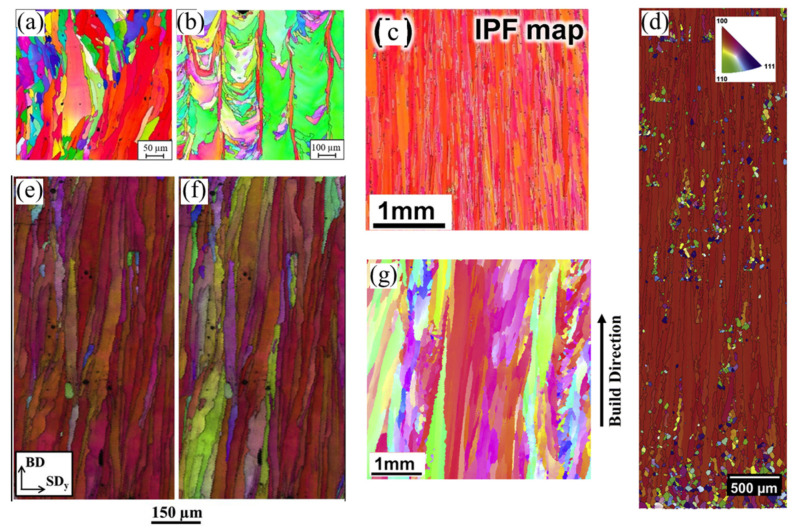
Examples of PBF-manufactured columnar grain structures: (**a**) AA2024 aluminium alloy [72], (**b**) 316L steel [70], (**c**) Co–Cr–Mo alloy [78], (**d**,**g**) IN718 [66,67], and (**e**,**f**) high-silicon (6.9% wt. Si) steel [16].

Coarse columnar grains represent a ‘classic’ AM grain structure, typical for many materials (Figure 2), including titanium alloys [15,43,51,55,64], nickel-based alloys [6,34,45,65,66,67], steels [16,18,34,42,68,69,70,71], some aluminium alloys [72,73], copper and its alloys [74,75,76,77], cobalt alloys [44,78], etc. These alloy systems under PBF processing conditions are usually free of pronounced heterogeneous nucleation in front of the solid–liquid interface and solidify epitaxially. Their strong morphological and often crystallographic texture significantly affects the mechanical properties of AM components, resulting in their anisotropy [4,5,6,12,13,14,15,16,17,25,26,36,42,43,71,72].

Experimental studies (e.g., [18,79,80]) have reported anisotropic residual stresses in LPBF-produced metallic materials. Their anisotropy was suggested to be related to the layer-by-layer nature of the process, directional scanning by discrete tracks (fast and non-uniform melting and solidification rates) [80,81,82], which is about Type I stresses. To our knowledge, experimental research on Type II or intergranular stresses formed during AM is lacking in the literature due to measurement challenges [81,83].

Mechanical anisotropy in MAM, however, might be related to different reasons. Let us consider LPBF-produced aluminium alloys as an example. Tradowsky et al. [84] noted improved tensile properties for horizontally built AlSi10Mg samples, i.e., with the longest dimension printed perpendicular to the build direction (BD) in comparison with the samples built vertically (~25% more strength; ~65% more elongation). They attributed this anisotropic behaviour to voids and their specific orientation. Significant changes in the total elongation of Al-12Si samples built horizontally and vertically have been demonstrated by Liu et al. [85] (40% anisotropy in elongation). The horizontal samples showed higher elongation, yield, and tensile strength. Differences in tensile and yield stresses, however, were not so pronounced (8.2% and 12% anisotropy, respectively). In [85], the anisotropy was related to the melt pool pattern, and similar conclusions have been drawn in [86]. However, data on the process-induced material anisotropy, which are reported in the literature, are contradictory. For instance, Ch et al. [87] observed opposite strength trends based on build orientation (~12% more strength for a vertically built AlSi10Mg); the percentage elongation was marginally higher for horizontally built samples. Prashanth et al. [88] studied LPBF-produced Al-12Si samples loaded at different angles to the BD and concluded on no dependence of the yield stress on the sample orientations. More details about the anisotropy of mechanical properties in AM Al alloys are given in [46].

Mechanical anisotropy in AM parts might represent a significant concern, particularly when designing and manufacturing components for high-performance or safety-critical applications. For instance, AM parts designed without considering anisotropy can exhibit pronounced performance variability depending on the printing orientation. Mechanical anisotropy can impose design and manufacturing constraints. Components expected to bear complex loadings might need to be redesigned to ensure uniform strength. Additional post-processing is often required to mitigate the anisotropy effects, which increases manufacturing time and costs. Understanding these implications is crucial for the broader adoption of MAM in various industries and would require increased training for designers, engineers, and operators to ensure they are equipped to make informed decisions during the design and manufacturing phases. In their seminal work, DebRoy et al. [13] pointed out the lack of comprehensive understanding of process-induced anisotropy’s impact on product performance. This highlights the need for further research in this area, particularly when a product is subjected to complex loading conditions.

**Figure 3:**
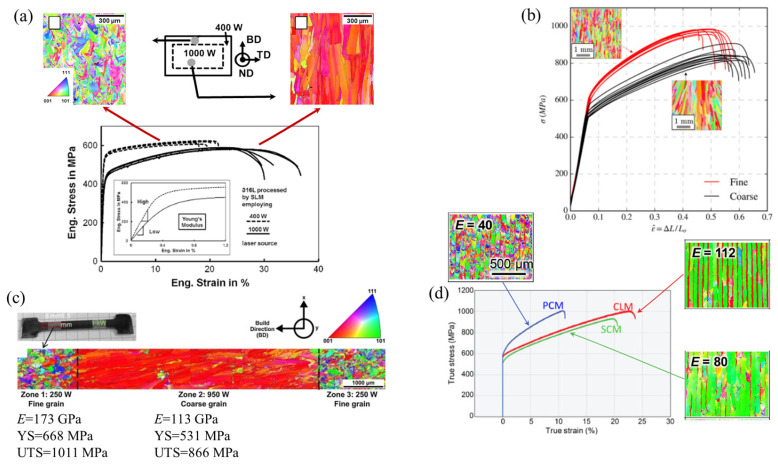
Examples of the impact of grain structure on the mechanical properties of PBF-manufactured alloys: (**a**) 316L austenitic steel [71], (**b**) Haynes 282 nickel-base superalloy [5], and (**c**,**d**) IN718 nickel-base superalloy [6,89]. *E* denotes Young’s modulus, with other abbreviations standing for yield strength (YS) and ultimate tensile strength (UTS). In subfigure (**d**), PCM stands for the polycrystalline microstructure; CLM stands for the crystallographic lamellar microstructure; and SCM stands for the single-crystal-like microstructure.

On the other hand, AM technology is known for its flexibility related to a number of process parameters that can vary for each build job. In fact, more than 130 parameters can affect the LPBF process and produced parts [9]. This flexibility underlies the technology potential for materials engineering and property tailoring. MAM with different process parameters can yield parts characterised by different microstructures and, thus, by different mechanical properties (Figure 3). For example, employing 400 W and 1000 W laser systems for printing 316L steel, Niendorf et al. [71] produced a weakly-textured fine-grained solidification structure and a highly-textured columnar grain structure with grains of more than 1 mm (Figure 3a). The former material was characterised by high Young’s modulus and strength while Niendorf and colleagues reported a decrease of Young’s modulus by a factor of 2 for the latter, highly textured material, which is very untypical for steels. Varying laser speed and power, Gokcekaya et al. [6] engineered a variety of grain structures in IN718, which were characterised by different morphologies and textures, including a single-crystal-like microstructure (SCM) with a Goss texture, a crystallographic lamellar microstructure (CLM) combining strong Goss and weak cube texture components, and a polycrystalline microstructure (PCM) with a weak orientation (Figure 3d).

### 2.3. Cellular–Dendritic Substructure

**Figure 4:**
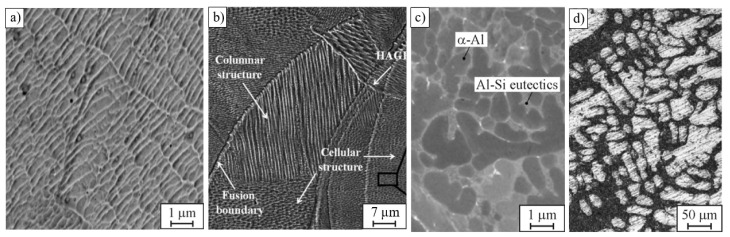
Examples of subgrain structures produced by additive and formative manufacturing: (**a**,**b**) as-built 316L stainless steel [69,90]; AlSi10Mg alloy produced by (**c**) LPBF [91] and (**d**) casting [92]. Subfigure (**b**) reveals the cellular and columnar substructures, fusion boundaries, and high-angle grain boundaries of LPBF 316L steel.

AM materials usually have a very fine sub-grain structure resulting from rapid solidification [3,4,6,16,18,42,43,45,48,49,50,51,52]. These closely oriented cells together create a single grain surrounded by high-angle grain boundaries, illustrated in Figure 4b. As an example, scanning electron microscopy (SEM) micrographs of as-built 316L stainless steel fabricated by LPBF are presented in Figure 4a,b. To compare microstructures produced by additive and formative manufacturing, Figure 4c,d show the cellular–dendritic and dendritic structures of AlSi10Mg alloy produced by LPBF and casting, respectively. The dendritic cell size of the cast AlSi10Mg alloy is by an order of magnitude larger than that of the AM material of the same composition (cf. Figure 4c,d).

The microstructural features, including a fine cellular structure, a dense network of cell boundaries, non-equilibrium phases, precipitates, etc., vitally affect the mechanical properties of PBF-fabricated materials, as compared to alloys manufactured conventionally [3,42,43,48,49,58,68,90,91,92,93,94,95,96]. A number of authors have claimed PBF-fabricated stainless steels to have superior yield stress, hardness, and/or ultimate tensile strength (UTS) [18,68,90,94,95,96,97]. The reported values show an increase in the yield strength and UTS from 220–270 MPa and 520–680 MPa for wrought stainless steels [97], respectively, to 350–600 MPa and 480–800 MPa for additively manufactured ones, respectively [42]. The enhanced strength is attributed to small dendritic cells that act as barriers to the dislocation motion [18,90,95]. In cast Al-Si alloys, needle- or plate-like Si structures result in localised shearing during the onset of plastic deformation and then in the formation and propagation of cracks, thereby deteriorating mechanical properties [3,58]. In contrast, LPBF-produced Al-Si alloys contain spherical nano-sized Si particles, which are characteristic of eutectic regions. These particles provide resistance against local shearing forces, which consequently suppresses crack initiation and propagation and thus improves strength [3]. According to Delahaye et al. [48], LPBF AlSi10Mg exhibits ductile fracture due to decohesion between Si particles and the aluminium matrix, which tends to occur at melt pool boundaries.

## 3. Process-Structure-Properties-Performance Concept

**Figure 5:**
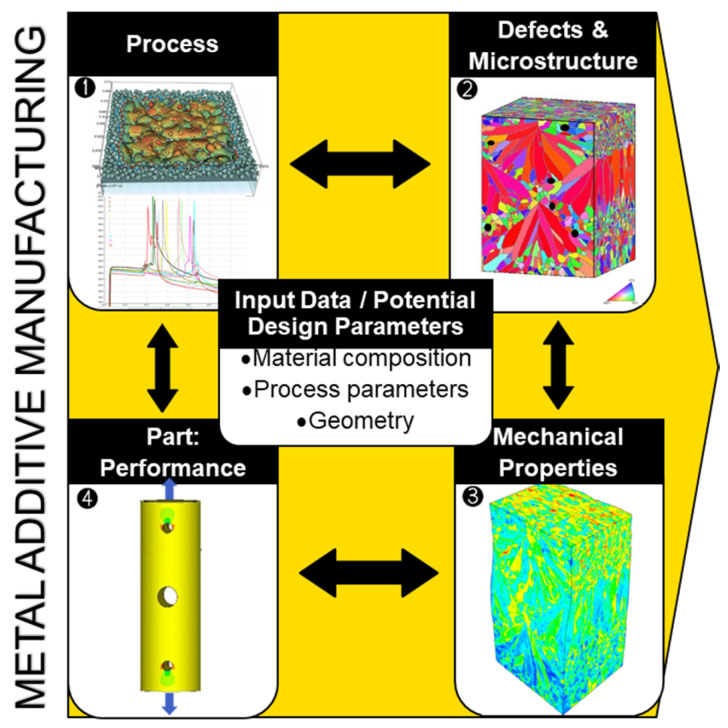
Integrated process–structure–property–performance modelling concept. The images are adapted from [29,118].

The concept of integrated PSPP modelling (Figure 5) links the manufacturing process with the structural performance of parts through the knowledge of the material microstructure and mechanical properties. This concept is an essential integral part of the progress of design and manufacturing. It forms the foundation of integrated computational materials engineering (ICME) which aims at the optimisation of materials development. Establishing the PSPP linkage for AM materials becomes imperative in the realm of a holistic computational design approach for AM [98]. This is particularly important for new materials enabled by technology, including functionally graded materials [99,100,101], metamaterials [102,103], and bio-inspired materials [104,105]. This linkage serves a dual purpose—it is vital for evaluating the performance of a particular component and for tailoring its microstructure to suit a specific application. Many authors (e.g., [2,20,23,32,40]) support the idea of the PSPP approach’s potency for a deep fundamental understanding of the deformation behaviour of a material under study, in addition to the two reasons mentioned above (certification and property tailoring). Global standards organisation ASTM International, formerly the American Society for Testing and Materials, considers ICME as the key element in the future state of AM qualification [106]. It is worth noting that despite active discussions on PSPP modelling for MAM, a very limited number of studies conduct actual research within this concept framework [17,28,32,107,108,109].

When discussing the PSPP and ICME concepts, it is worth highlighting the key role of multiscale modelling as both paradigms have arisen from the enhanced computing capabilities coupled with the development of new models. Many books on multiscale modelling discuss numerical methods and summarise models and approaches employed in PSPP and ICME to describe processes across different scales, involving from ground-state and molecular dynamics to continuum scale concepts (e.g., [110,111,112,113]). Enabling lattice structure printing, AM brought additional scale to multiscale modelling, which now separately considers a unit cell, i.e., a representation of perfectly periodic structures [98]. Homogenisation discussed in Section 1 forms the basis of multiscaling. We believe that the classical homogenisation approaches, where an RVE is applied to the whole macroscale part as a statistically sufficient representation of the material microstructure/behaviour, should be revised in the context of tailoring local properties with AM or of producing new technology-enabled hierarchical materials. Some of the smaller scale formulations, which contribute to the AM PSPP chain bridging the microscale with the macroscale, are outlined in this review, with the focus placed on the micromechanical description of AM materials. Accurate bridging strategies between scales for multiscale MAM simulations is a separate challenge to discuss, which is outside of the scope of this review, however.

Despite significant progress, multiscale modelling for MAM is still in its infancy for real world applications [114]. Amongst the noteworthy examples of how multiscale modelling has been used so far to improve the design and manufacturing of AM materials are a gearbox mount for an electric race car [115] and turbine blades [116]. Lammens et al. [115] applied the PSPP concept to guide the informed design and manufacturing of the gearbox mount. Their modelling approach included topology optimisation of the part geometry, distortion simulations, porosity estimations, microstructure simulations, and the MAM-aware fatigue prediction approach. Leveraging synergy between experiments and simulations, Wimler et al. [116] showed the potential to attain a wide range of material properties for an EPBF-fabricated multiphase γ-TiAl by changing line energy, which might be pivotal for turbine blade manufacture. Multiscale modelling may help optimise support structures and part placement and minimise residual stresses and defects in printed components. To a limited extent, this approach was explored for a compliant cylinder, square canonical geometry built to answer America Makes project no. 4026.001 and automotive upright in [117]. In the meantime, the authors [117] highlight the lack of publications that ‘describe in detail a functioning multiscale modelling’. With further development and adept application of multiscale modelling, there is a potential for its usage for optimising process parameters to produce components that not only showcase outstanding performance but are also tailored for intended applications.

## 4. Microstructure Modelling in AM

As outlined in Section 2, the microstructure has a major impact on the mechanical properties of PBF-produced components. Thus, modelling specific microstructural features of a 3D printed component is crucial for an accurate numerical description of its mechanical properties and performance [2,32]. This section explores different approaches for generating model microstructures, covering both physically based and geometrically based methods and image reconstruction. There also exist macroscale approaches that generally describe the microstructure evolution in terms of phases [119], using, e.g., the Johnson–Mehl–Avrami–Kolmogorov (JMAK) model. These approaches adapted for MAM can also be considered in PSPP modelling frameworks and produce output for the mechanical or thermomechanical modelling of AM materials (e.g., [28,120,121]). However, as these models do not visualise estimated AM microstructures, they fall outside the scope of this review.

### 4.1. Physically Based Microstructure Modelling

Microstructures are predominantly determined by thermal processes in PBF, making their numerical description crucial for physically based microstructure modelling (Figure 5). Various models and simulation approaches have been proposed to understand and control PBF which represents a complex interplay of different physical processes and phenomena. Worthy of mention are the approaches enabling (i) powder bed micro modelling, i.e., generating the powder bed of individual particles with the use of the ‘rain’ (ballistic deposition) model or discrete element method [122,123,124], (ii) the description of as-built residual stresses and distortion by virtue of finite element (FE) simulations [124], (iii) robust FE or finite difference (FD) calculations of temperature fields with pure conduction models [125,126,127], or (iv) more complex and computationally heavier prediction of melt pool dynamics with computational fluid dynamics (CFD) models using finite volume or lattice Boltzmann methods [122,125,128,129]. The latter type embraces models of different mathematical and numerical complexity, which may consider advanced laser-material interaction (e.g., ray tracing [129,130]), have different assumptions on the molten surface behaviour, from flat surface assumption [131] to integrated surface tracking algorithms [122,129], etc. More detail on temperature modelling for MAM can be found in comprehensive reviews [125,132].

However, some physical phenomena are still neglected in MAM process modelling. For example, Bayat et al. [125] highlight that most CFD models still neglect denudation. This phenomenon results in powder dispersion and thus causes process-induced surface finish. While neglecting denudation in the bulk of a part being built might be acceptable, it can cause significant discrepancies when modelling the top surface of a final layer. Furthermore, the scale at which heat transfer occurs (given the localisation of PBF AM) and the scale of the part being built can differ substantially. Bridging these scale differences in modelling is non-trivial. Balancing various multiphysics phenomena inherent in AM simulations with the need for efficient multitrack and multilayer simulations poses another challenge. Too much detail can make simulations computationally prohibitive while oversimplification may neglect critical behaviours, which highlights the delicate trade-off required for accurate and feasible thermal modelling and the need for the expert application of high-performance computing.

**Figure 6:**
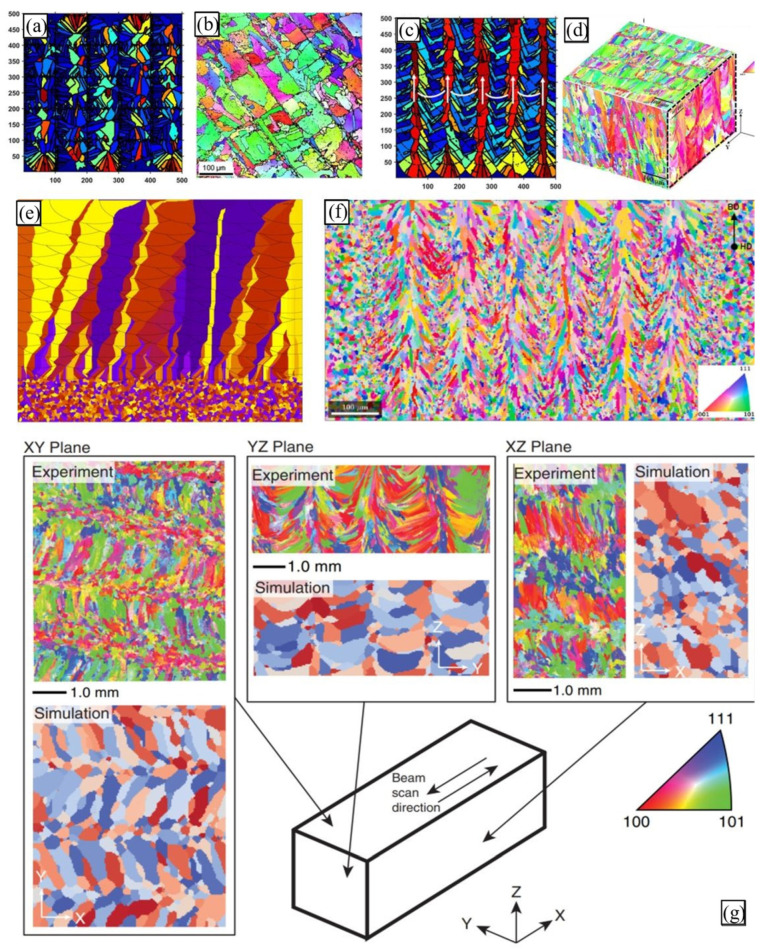
**Model-predicted grain structures of PBF-fabricated alloys**: CA-simulated grain structures produced using (**a**) cross-hatching and (**c**) uni-/bidirectional scanning patterns [137] compared with (**b**,**d**) experimental microstructures [143,144]; (**e**) simulated grain structure of selectively laser melted steel [138]; (**f**) inverse pole figure maps of the simulated LPBF AlSi10Mg alloy corresponding to <uvw>||hatch direction (HD) [139]; and (**g**) comparison between simulated and experimental microstructures of additively manufactured Inconel 718 [140,145].

Calculated temperature fields can serve as an input for modelling the microstructural features of metallic materials solidified during PBF (Figure 5). Common methods for this purpose are the phase-field (PF) approach [133,134,135,136], cellular automata (CA) [17,32,66,108,109,126,137,138,139], and the kinetic Monte Carlo (kMC) method [107,127,140]. Some of the model-predicted microstructures are illustrated in Figure 6.

Let us illustrate a few studies, while a detailed discussion of the existent microstructure modelling techniques for AM can be found in [98,141]. Akram et al. [137] implemented a 2D CA-based microstructural model to investigate grain evolution during PBF. Considering uni-, bidirectional, and zigzag with cross-hatching (island) scanning patterns, the authors demonstrated differences in grain shapes relative to a cross-section and the scanning strategy considered (Figure 6a–d). Earlier, Zinoviev et al. [138] developed a 2D cellular automata-finite difference (CAFD) model to describe the grain structure evolution observed during LPBF (Figure 6e). Later, the authors extended this model to the 3D case to describe the microstructure evolution in LPBF-produced Ti-6Al-4V alloy and 316L stainless steel [17,126,142]. Mohebbi and Ploshikhin [139] applied the same CA approach in simulations of an AlSi10Mg microstructure under LPBF conditions (Figure 6f). Rodgers and colleagues [71] described the 3D grain structure evolution during PBF and directed energy deposition by employing the Potts kMC model (Figure 6g).

In MAM modelling, the CA and kMC methods are employed for grain-scale simulations while the PF approach is applied to both the grain and substructure scale and is reported to be the most used method for microstructure simulations for MAM [98]. The PF method provides a high level of physics fidelity for the description of complex solidification morphology and the evolution of composition. However, applying this method requires profound expertise in the field due to uncertainty related to a number of parameters required for PF modelling and the very mathematics behind the PF model. The advantage of the CA and kMC approaches lies in their computational efficiency as compared to PF calculations performed at the same length scale. The grain-scale CA and kMC approaches are capable of simulating 3D virtually printed samples which consist of multiple layers and are made by multiple hatches within each layer [17,107,126,139,140,142]. The grain-scale PF simulation is limited by few tracks and a couple of layers in 3D [135], which is difficult to consider as a representative volume for a PBF-produced sample. The drawbacks of the kMC Potts approach as compared to the CA and PF methods include its inability to account for crystallographic orientations of grains and thus predict the material’s texture in the majority of implementations along with its failure to reproduce the correct mechanism of the grain structure evolution during metal AM [98,141].

### 4.2. Geometrically Based Microstructure Modelling and Image Reconstruction

**Figure 7:**
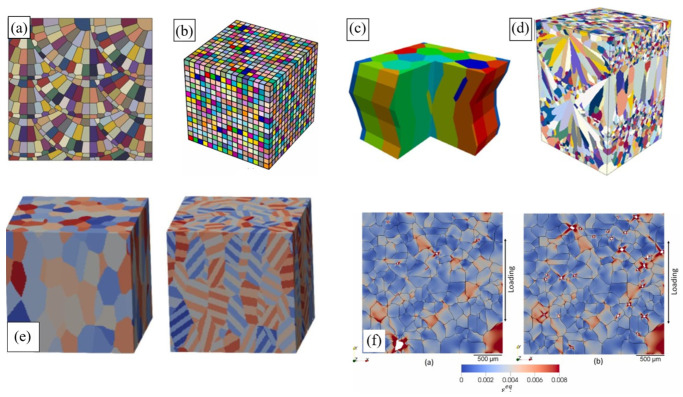
Synthetic grain structures of PBF-fabricated alloys: (**a**,**b**) microstructures of LPBF 316L steel [146,147] and (**c**) IN718 [31], which were generated using Voronoi tesselation; (**d**) polycrystalline model of an LPBF AlSi10Mg alloy simulated by SSP [29]; (**e**) RVEs of LPBF Ti-6Al-4V containing prior *β* grains without and with lamellar martensite *α’* microstructure [36], and (**f**) 2D domains combining synthetic grain structures with pores obtained using image reconstruction [148]. The microstructures in (**e**) and (**f**) were generated using Dream.3D.

**Figure 8:**
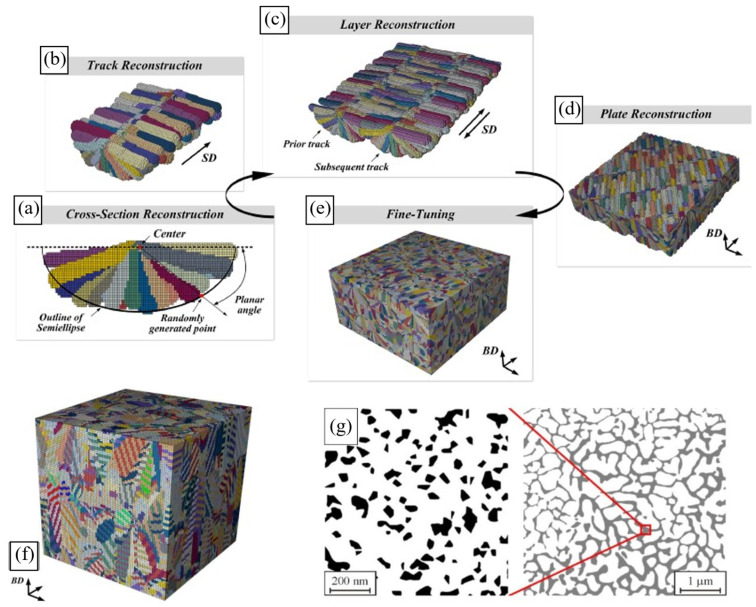
Grain structures of PBF-fabricated alloys reconstructed from experimental data. Subfigures (**a**–**e**) illustrate the step-by-step process of image reconstruction for grains of IN625 [152], including (**a**) the cross-section reconstruction, (**b**) hatch reconstruction, (**c**) layer reconstruction, (**d**) reconstruction of several layers, and (**e**) tuning. Reconstructed microstructures of (**f**) Ti-6Al-4V [152] and (**g**) Al-Si alloys [154].

The advantage of physically based microstructure modelling lies in the linkage between the AM process and the material’s internal structure, allowing for the description of process dynamics. In other words, we can digitally change the process parameters and obtain a different microstructure as a result of these changes. However, physically based methods are computationally demanding due to the need to simulate the printing process and the huge number of parameters to be calibrated. Alternatives include geometrically based microstructure modelling and image reconstruction which are showcased in Figure 7 and Figure 8.

An efficient option is to generate synthetic microstructures that statistically match real PBF-produced ones by the geometrical characteristics of microstructural elements. The synthetic microstructure generation approaches include spatial tessellation methods (e.g., Voronoi tessellation [31,146]) and semi-analytical packing tools (e.g., the step-by-step packing (SSP) method [29,30,149] or the Dream.3D packing algorithm [150]). While these methods are not physically based, they can still be successfully utilised in micromechanical simulations of AM materials [29,30,31,146,151]. Some examples of synthetic AM grain structures are illustrated in Figure 7.

Spatial tessellation creates polycrystals composed of convex polyhedra, with their shape and spatial arrangement dependent on the initial seed distribution (e.g., Voronoi tessellation-based models in Figure 7a–c). Some drawbacks of these algorithms include ‘non-physical’ planar boundaries between microstructural elements and challenges in statistically relating a synthetic microstructure to an experimental one, especially highly heterogeneous ones (e.g., LPBF Al-Si alloys or Scalmalloy^®^). Being statistics/empirically guided, packing tools enable designing 3D microstructure models of variable complexity and with a wide variety of geometrical features (Figure 7d–f). The packing concept implies filling a discretised computational domain with microstructural elements in a stepwise manner following geometrically based algorithms which are specifically defined for the microstructure type to be generated [149]. Compared to the tessellation-based approaches, the packing method can provide a more profound and realistic microstructure representation [149].

Finally, experimental image reconstruction is worth noticing as the approach that provides the most realistic description of the microstructure and defects [148,152,153,154,155]. Being easily implemented for 2D microstructures, these methods imply a rather complicated reconstruction process in the 3D case, however, with one of the pathways shown in Figure 8a–e. Figure 8 demonstrates examples of the generated microstructural models for AM IN625, Ti-6Al-4V, and AlSi10Mg alloys.

## 5. BVP Formulation for Microstructure-Based Mechanical Simulations of Additively Manufactured Metallic Materials

### 5.1. FE Implementation of a Boundary-Value Problem

Micromechanical simulations with an explicit incorporation of microstructural features imply a numerical solution to a BVP formulated in partial derivatives. The input data for these simulations come from microstructure models, which are discussed in Section 4. In an idealised case, grains can be represented by square/cuboid clusters (Figure 7b) [147,156,157], where each is related to a material point or a Fourier point depending on the numerical technique in use.

The governing equations of continuum mechanics, including the equations of motion or equilibrium, are applicable to any kind of materials and detailed in a large number of papers. Let us address here only the main issues related to numerical implementation using the finite element method (FEM). In contrast to the FD approximation applied to the partial differential equations (PDEs) in a strong form, FEM implies a transition to a weak integral form based, e.g., on the virtual work principle. The integral governing equations are then substituted by a set of algebraic equations resolved on an FE mesh. Both in quasistatic and dynamic simulations the load is incrementally applied to the RVE boundaries through kinematic and/or traction boundary conditions. Accordingly, the displacement and stress and strain fields in the rest of the computational domain are updated at the end of each increment to balance the load.

The time integration methods are divided into implicit and explicit ones. The implicit schemes, where unknown quantities appearing in the equations are calculated through some values which are also unknown at the beginning of the time increment, can be used in solving both static and dynamic BVPs. The FE equations of static equilibrium can be written as:

(1) $$\left[K\right]\left\{u\right\}-\left\{f\right\}=0.$$

Here, $\left\{f\right\}$ and $\left\{u\right\}$ are the nodal force and displacement vectors, respectively, and $\left[K\right]$ is the global stiffness matrix. For brevity, bold-face symbols are utilised to represent tensors or dyads in the text, with their order being inferred from the context. The numerical solution to Equation (1) involves calculating the inverse of $\left[K\right]$ which along with the iteration procedure requires substantial computational resources.

With the explicit solver, the unknown quantities appearing in the PDEs are expressed through the parameters known from the previous time step. These schemes are applicable to the solution of a dynamic BVP alone. The equation of motion in the matrix form is:

(2) $$\left[M\right]\left\{\ddot{u}\right\}=\left\{f\right\}-\left[K\right]\left\{u\right\},$$

where $\left[M\right]$ is the lumped mass matrix, which is easily inverted, and the dot shorthand notation denotes the time derivative. Each step of an explicit calculation requires neither iteration nor matrix inversion and, thus, is much less computationally consuming than that of an implicit solver. The drawback of the explicit schemes is their conditional stability. It imposes stringent limitations on the value of the time increment to ensure that the distance that the stress wave covers within a time step does not exceed the smallest mesh step. As a rule, a stable time step is too small to simulate long-time quasistatic processes unless the load velocities are artificially increased. In the latter case, certain computational tricks are necessary to obtain a quasistatic solution [35,158]: (i) the load velocity must be increased smoothly to minimise the acceleration term in the equation of motion and (ii) the constitutive equations should not be strain rate dependent.

### 5.2. Kinematics and Constitutive Laws

The system of governing equations is completed with kinematics, which relates the strain and displacement fields, and with constitutive laws, which relate the stress and strain tensors to specify the material response. In the general case of finite strains, a multiplicative decomposition of the total second-rank deformation gradient tensor $F=x_{i,j}$ into elastic $F^{e}$ and plastic constituent parts $F^{p}$ is used.

(3) $$F=I+\frac{\partial u}{\partial x^{0}}=F^{e}F^{p}.$$

Here, $J\equiv \det\left(F\right)=det\left(F^{e}\right)>0$; $det\left(F^{p}\right)=1$; I is the identity matrix; u is the displacement field; superscripts e and p stand for the elastic and plastic parts, respectively; and $x_{i,j}=\partial x_{i}/\partial x_{j}^{0}$, where the vector $x_{j}^{0}$ defines the initial/undeformed/relaxed reference configuration and the vector $x_{i}$ defines the deformed current configuration. The superscript 0 refers to the initial configuration.

The elastic strain can be defined in terms of the Green–Lagrange strain tensor as

(4) $$E^{e}=\frac{1}{2}\left(C^{e}-I\right)=\frac{1}{2}\left({F^{e}}^{T}F^{e}-I\right).$$

where $C^{e}$ is the right Cauchy–Green elastic deformation tensor and ${F^{e}}^{T}$ stands for the transpose of $F^{e}$. This brings us to the generalised Hooke’s law yielding the second Piola––Kirchoff stress tensor $S^{e}$ (applicable to scenarios involving large rotations and small elastic strains)

(5) $$S^{e}=\tilde{C}:E^{e}=\tilde{C}:\frac{1}{2}\left({F^{e}}^{T}F^{e}-I\right),$$

(6) $$S^{e}=J{F^{e}}^{-1}\sigma {F^{e}}^{–T}.$$

Here, $\tilde{C}$ is the fourth-order elastic stiffness tensor; σ is the Cauchy stress tensor; and ${F^{e}}^{-1}$ is the inverse of $F^{e}$.

In the case of small strains, it is acceptable to use additive decomposition of total strain ε into elastic $\varepsilon^{e}$ and plastic parts $\varepsilon^{p}$:

(7) $$\varepsilon =\varepsilon^{e}+\varepsilon^{p},$$

which transfers to strain rates as:

(8) $$\dot{\varepsilon }={\dot{\varepsilon }}^{e}+{\dot{\varepsilon }}^{p}.$$

The generalised Hooke’s law can thus be formulated as:

(9) $$\dot{\sigma }=\tilde{C}:\left(\dot{\varepsilon }-{\dot{\varepsilon }}^{p}\right).$$

### 5.3. Constitutive Models of the Plastic Behaviour of Grains

Selecting an appropriate constitutive model (CM) is crucial for the reliability of computational results. There are different types of CMs, and the choice largely depends on the material and research objectives. The simplest solution disregards crystal lattice effects, relying on macroscopic stress–strain curve approximation to describe grain behaviour. In such models, the plastic response of individual crystals is typically represented in terms of isotropic plasticity and the material heterogeneity is reflected through a certain variation of material properties relative to their macroscopic values [156,159,160]. For example, describing the mechanical behaviour of friction-stir-welded aluminium, Balokhonov et al. [159] randomly varied elastic shear and bulk moduli from grain to grain, with a minimum-to-maximum value difference of 20%. This value can be determined from hardness testing. The yield stress in [159] was defined according to the Hall–Petch relation. Pham et al. [160] considered a material inhomogeneous parameter $\kappa_{i}\in \left[0.1;2.5\right]$ to define flow stresses for different groups of grains of austenite steel, $\sigma_{i}=\kappa_{i}\sigma_{Y}$. Here, $\sigma_{Y}$ follows a Voce-type law, $\sigma_{Y}=\sigma_{0}+Q\left[1-\exp\left(-b{\bar{\varepsilon }}^{p}\right)\right]+H{\bar{\varepsilon }}^{p}$; $\sigma_{0}$ stands for the initial value of yield strength; Q is the saturation value; b denotes the rate of saturation; H is the slope of the linear term added to prevent excessive strain localisation from the stress saturation; and ${\bar{\varepsilon }}^{p}$ represents the equivalent plastic strain. Furushima et al. [156] introduced the material inhomogeneity parameter to Hollomon’s classical constitutive equation [161], $\sigma =\kappa F\varepsilon^{n}$, in order to model the uniaxial and bi-axial tension of polycrystalline A5052-O. Here, σ and ε stand for the equivalent stress and strain, respectively; F and n are the material’s constants determined in uniaxial tensile tests. The idea of the material inhomogeneity parameter becomes attractive on a higher spatial scale and is particularly relevant for functionally graded materials [162]. The latter have recently gained significant attention due to the progress in the metal AM space which enabled the manufacture of complex functionally graded structures with precision along with new opportunities for designing and producing functionally graded materials that were previously challenging or impractical using conventional manufacturing methods.

The above-discussed CMs are commonly applicable to non-textured polycrystalline materials with an isotropic homogenised response. In this sense, they might be attractive for the microstructure-informed mechanical simulations of the novel fine-grained AM materials we learned to produce recently [163,164,165] or of functionally graded materials at a higher spatial scale [162], as discussed above. However, for many PBF-fabricated materials, which are commonly characterized by a strong crystallographic texture (see Section 2), these models may not provide a proper description and thus should be modified to take the effects of grain orientation into consideration. For example, in their microstructure-based CM, Zinovieva et al. [32] proposed to relate the yield stress of individual grains with their size and orientation as:

(10) $$\sigma_{ini}^{\left(g\right)}=\frac{\tau_{c}}{m_{max}^{\left(g\right)}}+\frac{k_{y}}{\sqrt{d_{g}^{\left(g\right)}}},$$

Here, $\tau_{c}$ stands for the critical resolved shear stress (CRSS); $m_{max}$ denotes the maximum value of the Schmid factor; $k_{y}$ is the grain boundary resistance; $d_{g}$ represents the grain size; and the superscript $\left(g\right)$ reflects the relation of a particular variable to a grain. The second term on the right accounts for the Hall–Petch effect. Strain hardening was taken into account in a phenomenological way, as a function of the yield stress on the accumulated equivalent plastic strain:

(11) $$\sigma_{y}^{\left(g\right)}=\sigma_{ini}^{\left(g\right)}+A\left[1-\exp\frac{\varepsilon_{eq}^{p}}{\varepsilon_{r1}^{p}}\right]+B\left[1-\exp\frac{\varepsilon_{eq}^{p}}{\varepsilon_{r2}^{p}}\right].$$

Here, A and B denote the fitting parameters; $\varepsilon_{eq}^{p}$ is the equivalent plastic strain; and $\varepsilon_{r1}^{p}$ and $\varepsilon_{r2}^{p}$ are its reference values.

A more accurate description of PBF-produced materials calls for crystal plasticity (CP) models. They explicitly take into account the crystalline structure and geometrical features of dislocation glide on active slip systems. These models are capable of reproducing the grain-scale anisotropy of mechanical properties, plastic strain localisation, and stress concentration, provided that the grain structure is considered explicitly. Not long ago, this area attracted interest from the modelling community, which is illustrated by the very recent growth of publications in the field. According to Web of Science, 46 papers have been published since 2017, with 23 publications issued since 2022 (sum of the search queries ‘powder bed fusion’ or ‘selective laser melting’ or ‘selective electron beam melting’ or ‘direct laser metal sintering’ and ‘crystal plasticity’ and ‘simulations’). However, their number still remains rather low and thus there exists a wide space for the development of micromechanical models for AM and their applications to numerical experiments.

**Figure 9:**
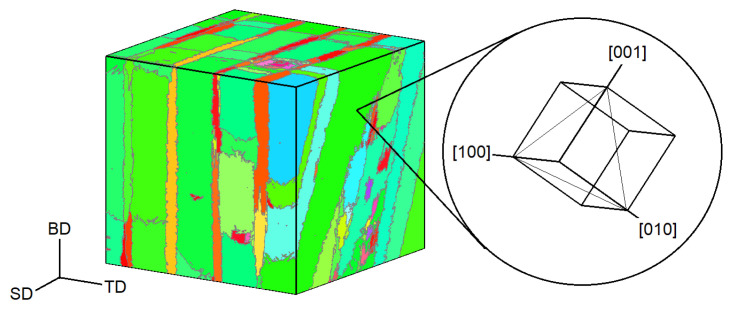
Schematic representation of an LPBF-fabricated polycrystalline model microstructure showing global (sample) and local (crystal) frames. The global coordinate system refers to the build, scan, and transverse directions (BD, SD, and TD, respectively) [166].

Within the CP concept, a polycrystal is thought of as an ensemble of grains, each with its crystallographic orientation relative to a global frame (Figure 9). Numerically, each grain is assigned a local coordinate system which is associated with the crystal lattice.

In crystalline materials, plastic deformation occurs on well-defined slip systems that are typical to the crystallographic lattice. The geometrical equations

(12) $$L^{p}={\dot{F}}^{p}{F^{p}}^{-1}=\sum_{\alpha }{\dot{\gamma }}^{\left(\alpha \right)}{Z_{s}^{0}}^{\left(\alpha \right)}$$

relate the plastic part to shear contribution ${\dot{\gamma }}^{\alpha }$ on active slip systems α. Here, $Z_{s}^{0}=s_{s}^{0}⊗n_{s}^{0}$ represents the Schmid tensor that describes the slip system geometry and orientation in the crystal coordinate system. Unit vectors $s_{s}^{0}$ and $n_{s}^{0}$ are associated with the slip direction and slip plane normal. $Z_{s}^{0}$ refers to the relaxed configuration; however, it may be related to the current configuration as $s_{s}=F^{e}s_{s}^{0}$ and $n_{s}={F^{e}}^{–T}n_{s}^{0}$ [167] or as $Z_{s}=$ ${F^{e}}^{–1}{Z_{s}^{0}F}^{e}$ [168]. Some researchers relate the right part of Equation (12) to the current configuration of the Schmid tensor [168].

Depending on the material under consideration, the description of twinning might be introduced in Equation (12) in a similar fashion as adding $\sum_{\beta }{\dot{\gamma }}_{tw}^{\left(\beta \right)}{Z_{tw}^{0}}^{\left(\beta \right)}$ [168,169]. Here, $Z_{tw}^{0}=s_{tw}^{0}⊗n_{tw}^{0}$ represents the tensor that defines twinning, $s_{tw}^{0}$ and $n_{tw}^{0}$ are the twin Burgers direction unit vector and the twin plane normal unit vector, respectively.

The slip rate $\dot{\gamma }$ for any slip system α can be described by physically based or phenomenological models which relate the slip rate to the resolved shear stress. Some examples include a Hutchinson-type power law that omits [29,30,148,170] or takes into account back stress $\chi^{\left(\alpha \right)}$ [171,172]; see Equation (13) vs. Equation (14), respectively:

(13) $${\dot{\gamma }}^{\left(\alpha \right)}={\dot{\gamma }}_{0}{\left|\frac{\tau^{\left(\alpha \right)}}{\tau_{c}^{\left(\alpha \right)}}\right|}^{\nu }sgn\left(\tau^{\left(\alpha \right)}\right),\;sgn\left(x\right)=\left\{\begin{array}{c} -1,\;\;x<1\\ 1,\;\;x\geq 1 \end{array}\right.,$$

or

(14) $${\dot{\gamma }}^{\left(\alpha \right)}={\dot{\gamma }}_{0}{\left|\frac{\tau^{\left(\alpha \right)}-\chi^{\left(\alpha \right)}}{\tau_{c}^{\left(\alpha \right)}}\right|}^{\nu }sgn\left(\tau^{\left(\alpha \right)}\right),\;sgn\left(x\right)=\left\{\begin{array}{c} -1,\;\;x<1\\ 1,\;\;x\geq 1 \end{array}\right.,$$

or a Norton-type law [167,173]

(15) $${\dot{\gamma }}^{\left(\alpha \right)}={\langle \frac{\left|\tau^{\left(\alpha \right)}-x_{s}^{\left(\alpha \right)}\right|-r_{s}^{\left(\alpha \right)}}{K}\rangle }^{\nu }sgn\left(\tau^{\left(\alpha \right)}-x_{s}^{\left(\alpha \right)}\right)$$

Here, ${\dot{\gamma }}_{0}$ stands for the initial shear strain rate; τ is the resolved shear stress; $\tau_{c}$ is the CRSS; $x_{s}$ and $r_{s}$ represent the hardening variables related to the kinematic hardening and isotropic hardening, respectively; and ν is the strain rate sensitivity coefficient. Variables ${\dot{\gamma }}_{0}$ and ν control the strain rate sensitivity. The back stress $\chi^{\left(\alpha \right)}$ is caused by dislocation interactions through elastic stress fields, which each dislocation forms. These interactions hinder plastic deformation and work harden the material. Some studies employ more complex, physically based models to include dislocation kinetics (e.g., [157,168,174,175]).

The resolved shear stress τ acting on the slip system α can be calculated by projecting the macroscale stress tensor to the slip system as follows:

(16) $$\tau =\sigma :Z_{s}=\left(C^{e}S^{e}\right):Z_{s}^{0}\approx S^{e}:Z_{s}^{0}.$$

The slip system becomes active when the resolved shear stress τ is equal to or greater than the CRSS $\tau_{c}$. The CRSS definition that properly accounts for strengthening mechanisms is a key factor for accurate prediction of the material’s mechanical behaviour.

### 5.4. Description of Hardening Mechanisms in PBF-Produced Materials

Strengthening mechanisms imply a combination of physical processes specific to each kind of material, including its processing which affects the microstructure. Determining a hardening function which properly describes these mechanisms is a challenging task in the case of PBF-produced materials. The more mechanisms are considered, the more parameters must be calibrated, which may significantly complicate numerical implementation. Researchers always have to find a compromise between computational cost, the complexity of numerical implementation, and the informativeness of the resultant output data.

Depending on the hardening function under consideration, the constitutive models can be categorised into two main groups. The first group includes phenomenological models which are based on developing a hardening law by the direct approximation of a stress–strain curve. These models are capable of simulating deformation processes in particular materials under particular loading conditions. Generally, such models are not predictive, although they can still be extended to other kinds of loading in some cases. The advantages of these models are their applicability in studying the stress–strain characteristics at the micro- and mesoscales at relatively low computational costs.

Another category covers physically based models that are not only descriptive but also predictive for stress–strain analysis in a wide range of loading conditions. However, such a possibility leads to a complex mathematical formulation, a large number of parameters that should be determined from independent experiments and calibrated, and high computational costs.

Generally, hardening functions are represented through a sum of contributions from various deformation mechanisms (dislocation gliding and twinning), impurity and precipitation effects, grain boundary strengthening, etc. As-built PBF alloys have been reported to demonstrate multiple strengthening mechanisms [176,177]. For instance, analysing the characteristics of the deformed LPBF AlSi10Mg, the authors [176,177] concluded that the yield strength of this alloy can be defined as:

(17) $$\sigma_{y}=\sigma_{0}+{∆\sigma }_{Orowan}+{∆\sigma }_{HP}+{∆\sigma }_{disl},$$

where $\sigma_{0}$ stands for the internal friction stress; ${∆\sigma }_{HP}$ is the term of stress contribution due to eutectic cell walls; the stress contribution ${∆\sigma }_{Orowan}$ is related to the Orowan mechanism because of the presence of Si precipitates; and ${∆\sigma }_{disl}$ is the term resulting from the dislocation hardening due to pre-existing dislocation network. The stress contribution by Orowan strengthening in an Al-Si binary system can be calculated as:

(18) $${∆\sigma }_{Orowan}=\frac{\varphi Gb}{d_{Si}}{\left(\frac{6V_{Si}}{\pi }\right)}^{1/3},$$

and the stress contribution by dislocation hardening can be estimated as:

(19) $${∆\sigma }_{disl}=\beta MGb\sqrt{\rho_{d}}.$$

Here, φ and β are the material constants; G stands for the shear modulus; b denotes the Burgers vector; $d_{Si}$ represents the diameter of Si precipitates and $V_{Si}$ is their volume fraction; M stands for the Taylor factor; and $\rho_{d}$ is the dislocation density.

Typically, hardening laws in both phenomenological and physically based models incorporate the initial yield stress and the strengthening mechanisms attributed to the Hall-Petch effect (due to grains or eutectic cell walls [176]), among other terms. Real art starts from the definition of these additional terms. Table 1 summarises various hardening laws considered in the micromechanical simulations of PBF materials.

## 6. Microstructure-Informative Mechanical Simulations of Additively Manufactured Metallic Materials

### 6.1. Two-Dimensional Microstructure-Based Mechanical Simulations

The first computational models incorporating PBF-produced microstructures explicitly used an essential idealisation of microstructural features or were mostly reduced to 2D approximations. Commonly, PBF AM parts are characterised by a directionally dependent microstructure with considerably different grain patterns observed in the sections perpendicular to the build, scan, and transverse directions (BD, SD, and TD, respectively). Thus, the applicability of 2D models which are capable of reproducing microstructural features in a single plane becomes rather questionable. Nevertheless, some successful attempts in 2D microstructure-based simulations of LPBF metals are worth noting.

Andani et al. [146,179] developed a semi-plane 2D grain model of an LPBF 316L steel to take into account the grain structure within several melt pools and layers in a specimen section perpendicular to a scan direction (TD-BD). A Voronoi tessellation algorithm was employed to reproduce the melt pool geometry and grain morphology (Figure 7a). The results, however, were not compared with experimental microstructures and failed to reproduce typical columnar grain structures observed in LPBF 316L steel (e.g., Figure 2b and Figure 3a). The grain behaviour was simulated within a conventional crystal plasticity finite element method (CPFEM) approach. A cohesive zone model was implemented to simulate crack nucleation and growth along the melt pool boundaries. This approach, while simplified, revealed important findings about pre-existing cracks in LPBF 316L steel drastically decreasing ductility and fracture stress [146]. They also emphasised that texture control improves ductility and reduces fracture stress in the LPBF parts. The authors indicated that reducing a hatch distance (which was indicated in the paper as a decrease in the melt pool size) had detrimental effects on the damage properties of the AM metallic parts. Note that this effect might be treated as purely numerical because reducing the hatch distance drastically affects the microstructure of AM materials [193,194,195]. The impact of grain orientation, loading direction, and hatch spacing on the mechanical properties of AM components was also addressed [179]. Unfortunately, the studies [146,179] lack the analysis of local stresses and strains.

**Figure 10:**
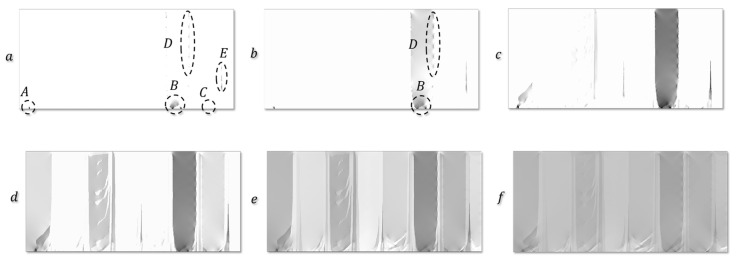
Equivalent plastic strain patterns in as-build LPBF 316L steel captured at the instants of plastic deformation labelled in Figure 11. The white-to-black greyscale corresponds to the degree of strain ranging from 0 to (**a**) 0.07, (**b**) 0.09, (**c**) 0.13, (**d**) 0.2, (**e**) 0.39, and (**f**) 1.57% [32].

**Figure 11:**
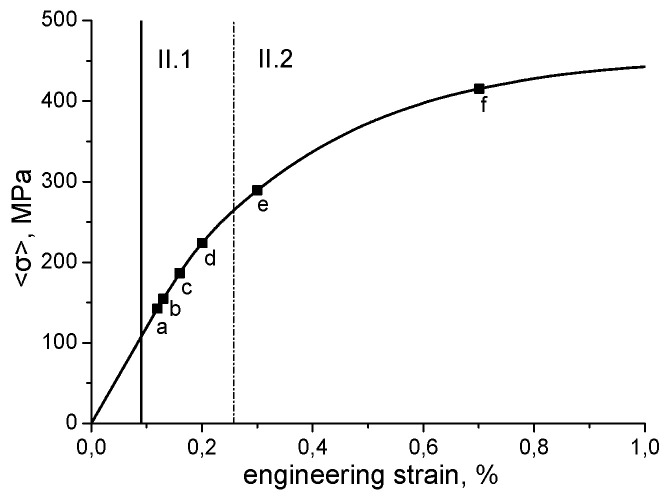
Stress–strain curve of an LPBF 316L sample loaded along the build direction [32].

Zinovieva et al. [32] calculated the mechanical response of a 2D grain structure formed on a TD-BD cross-section of an LPBF 316L steel sample. Following the concept of integrated PSPP simulations, the grain structure was computationally predicted using the physically based CAFD approach proposed in [138]. The grain behaviour was described by a phenomenological model which related the yield stress of individual grains with their size and orientation through Equations (10) and (11). The analysis of local stress and strain fields showed that the strain localisation regions were mainly formed in the bottom region of the LPBF model sample where the grain selection had not yet occurred, particularly along the boundaries of fine elongated grains (Figure 10 and Figure 11). The authors, however, highlighted the urgent need for 3D simulations to catch some missing effects.

**Figure 12:**
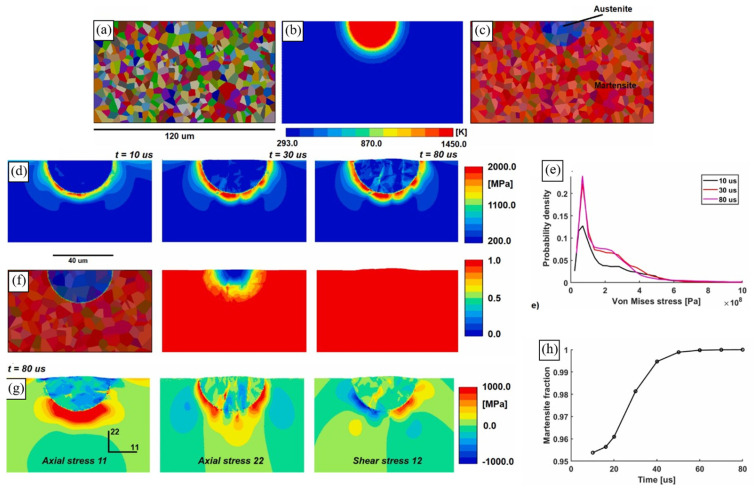
Microstructure-informed mechanical simulations for a laser surface remelted H13 tool steel: (**a**) model microstructure; (**b**) temperature distribution at simulation onset; (**c**) initial martensite (red) and austenite (blue) phase distribution and (**f**,**h**) its evolution; (**d**) evolution of von Mises stresses and (**e**) their probability plot; and (**g**) axial stresses and in-plane shear stress following the cooling process [175].

Lindroos et al. [175] presented 2D microstructure-based calculations of grain-scale residual stresses developing within an H13 tool steel weld under cooling. Their thermomechanical dislocation-based CP model depicted plastic deformation of austenite and martensite phases and thermomechanically induced solid-state phase transformations. The analysis revealed strongly inhomogeneous stress–strain distributions within the melt pool, with the highest stresses concentrating at the interfacial regions between the austenite and martensite phases along the melt pool boundaries (Figure 12). In the context of the examined subject matter, the authors discussed the salient features of experimental LPBF single-track microstructures and residual stresses emanating from the AM process. It is noteworthy, however, that within their computational framework, Lindroos et al. [175] reproduced laser surface remelting [196] rather than an LPBF single track. Moreover, the authors [175] chose to adopt a simplified representation of equiaxed grain structure which deviates significantly from microstructures observed in welds and MAM single tracks. The authors concluded on the influence of both crystal structure and grain orientation distribution on the local stress–strain patterns and indicated the importance of accounting for a realistic AM microstructure in micromechanical simulations. They also highlighted that the phase distribution creates pronounced stress concentration and the high density of cleavage opening stress near the melt pool boundary, while lower stresses characterise the bulk of the melt pool due to phase transformation acting against shrinkage.

Valuable as they are, these simulations were conducted in the plane strain or plane stress formulation. Thus, they are limited in terms of out-of-plane microstructural effects and might not be as accurate as full 3D simulations in capturing strain localisation and mechanical anisotropy due to neglecting the full 3D nature of the material’s microstructure and behaviour.

### 6.2. Three-Dimensional Mechanical Simulations for Synthetic Microstructures

Micromechanical simulations for synthetic 3D polycrystalline structures typical for AM metals have been reported in a number of papers. Notably, due to its computational efficiency, the crystal plasticity fast Fourier transformation (CPFFT) method [157] is becoming preferred compared with the conventional CPFEM solvers. In contrast to the implicit FEM, the FFT solver is much more computationally efficient as it does not require the global stiffness matrix to be stored during the iterative procedure.

Ozturk and Rollett [185] applied CPFFT to model periodic RVEs of grains generated using DREAM.3D software. The primary *β* grains elongated along the BD were further modified to account for the dual-phase microstructure which is formed upon the solid-state transformation and consists of a *β* matrix and *α* laths. Stress–strain partitioning between the hexagonal close-packed and body-centered cubic phases was analysed and the relationships between the phase volume fraction, size and morphology, and resulting mechanical response were established. While the study does not discuss local stress and strain distribution in detail, it concludes on the sensitivity of the deformation response to the fraction of *α*-phase and the texture of *β*-grains. Namely, the local deformation behaviour of an LPBF Ti-6Al-4V RVE with a lower *α*-phase fraction is controlled by both *α*- and *β*-phases. Conversely, when the RVE contains an increased α-phase fraction, it dominates the response, demonstrating higher local von Mises stresses as compared to the *β* matrix.

**Figure 13:**
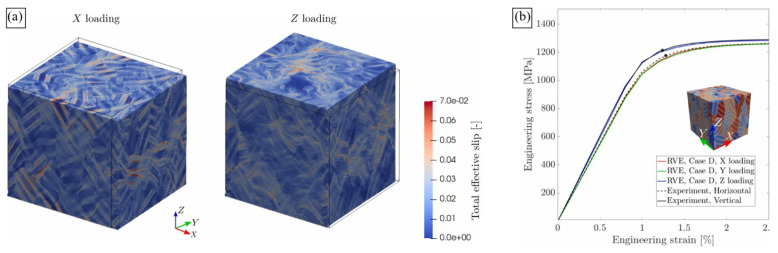
Microstructure-informed simulations of the uniaxial tension of the as-built LPBF Ti-6Al-4V in different directions: (**a**) total effective slip at 2% overall strain under loading along the X and Z directions, respectively and (**b**) macroscopic comparison of stress–strain curves obtained experimentally and numerically [36].

Later, Somlo et al. [36] employed the same computational approach to simulate the local and global deformation behaviour of LPBF Ti-6Al-4V RVEs composed of dual-phase grains (Figure 7e). The authors provided a thorough analysis of the contributions of different slip systems to plastic deformation and demonstrated the model’s ability to reproduce the anisotropy of mechanical response (Figure 13). Different synthetic textures were able to reproduce the experimentally determined elastic anisotropy with a reasonable degree of accuracy. However, the RVE that took into account the microstructure platelets arranged in two mutually perpendicular crystal orientations in each grain was shown to yield the most realistic elastic constants. The authors concluded on more the pronounced heterogeneity of the total effective slip distribution under loading in the X direction and on the smoother pattern when loaded in the Z direction (Figure 13a). High effective slip typically occurs close to the interface between *α*’-laths.

In spite of the authors’ efforts in summarising experimental observations on AM Ti-6Al-4V microstructures [36,185], a discernible discrepancy emerged with respect to the interpretation of the equiaxed prior *β* grain structure discussed in [197]. Gong et al. [197] expounded upon an equiaxed prior *β* structure observed within the scanning (SD-TD) plane from where it was concluded that prior *β* grains have a rod-like morphology. This, however, appears to have been misinterpreted by the authors [36,185] who considered 3D equiaxed prior *β* grains in their simulations. This additionally highlights the significance of 3D analysis for complex AM microstructures, which was discussed in [142]. Relying solely on the examination of isolated 2D cross-sections could potentially result in misleading interpretations of the microstructural characteristics. A pertinent example of this is evident in the misrepresentation of columnar grains as equiaxed when assessed from cross-sections perpendicular to their principal growth axis.

Tang et al. [152] presented a multiscale CPFEM framework aimed at evaluating the structure–property relationship of LPBF metals using 3D synthetic microstructures which were generated from experimental EBSD images. The microstructure generation procedure involved the packing of grains into a voxel-meshed cuboid volume to mimic a realistic grain morphology within multiple tracks and multiple layers (Figure 8a–e). The proposed approach was validated on the example of LPBF-produced Ti-6Al-4V subjected to uniaxial tension, compression, and cyclic loadings. The resulting grain model also took into account the presence of *α*’ martensite in primary *β* grains. The stress–strain curves obtained for synthetic microstructures agreed with those obtained experimentally; however, the grain-scale stress–strain fields were not analysed. Furthermore, it would have been beneficial to compare the model microstructure with those observed in the experiments performed to calibrate and validate the mechanical model rather than with the microstructure of selectively laser sintered Ti-6Al-4V which demonstrated *α*’ martensite only.

A substantial proportion of microstructure-based mechanical simulations have been carried out for LPBF Al-Si alloys. A complex hierarchical structure formed in these alloys during MAM crucially complicates the accurate prediction of their performance. According to extensive experimental data (e.g., [43,46]), the structural features affecting the deformation behaviour of the LPBF Al-Si parts are scaled from several nanometers to several millimetres. Apparently, explicit consideration of the whole spectrum of structural features within a single simulation is inapplicable. A common practice is to simulate the microstructural features and deformation events on a scale of interest on a stand-alone basis or to use a simplified representation of microstructure morphology.

Zhang and Andra [187] predicted the mechanical properties of an LPBF AlSi10Mg alloy at the micro- and macroscales using CPFFT simulations. A set of 3D RVEs were synthetically generated to take into account the geometry and orientations of Al grains, homogeneously distributed Si particles with a given effective aspect ratio, and pores with a prescribed volume fraction. The authors provided a rigorous parametric analysis of CP model parameter effects on the microscale stress partitioning between the Al and Si phases and the macroscopic stress. The calculations identified the highest von Misses stress in the Si particles. The authors, however, noted that such stress values might be unrealistic due to particle fracture or interfacial debonding. Although the microstructure morphology was represented in a simplified way, neglecting inhomogeneous Si distribution in space, the model was shown to capture the effects of Si particle aspect ratio and porosity on the macroscale stress–strain response in line with the experimental data [187]. An in-depth look into these effects on the local stresses and strains would have added value. The results highlighted the key role of the initial slip resistance $\tau_{0}$ in the macroscopic yield strength of the alloy and in the phase stresses at small applied strains and the primary role of the slip hardening parameters $h_{0}$, a, and $\tau_{s}$ (see Equation (26)) and the effective aspect ratio of Si particles in the alloy’s UTS and in the phase stresses at large applied strains.

**Figure 14:**
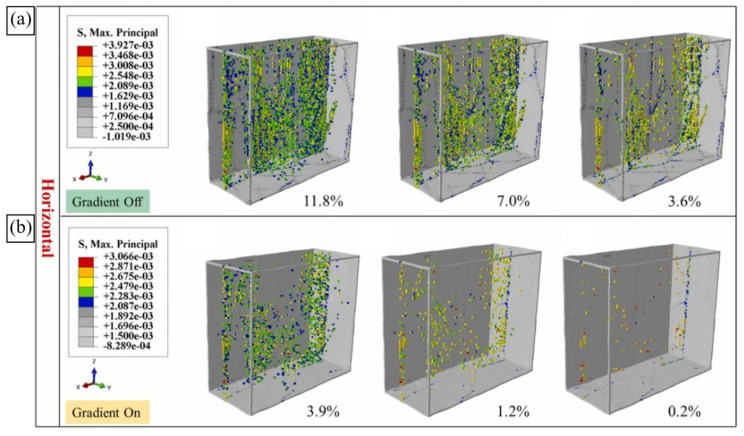
Microstructure-informed simulations of as-built LPBF AlSi10Mg (substructure scale) under uniaxial tension along X: results obtained with (**a**) a CP model and (**b**) a mechanism-based strain gradient CP model, which illustrate the effect of microscale strain gradients. Volumes describe the substructure observed in the bulk of the melt pool (fine melt pool zone). The colour highlights the maximum principal stress, which is higher than a threshold value at the strain of 0.1 [189].

The microscale stress–strain partitioning between the Al and Si phases was also addressed in [189]. Based on the experimental microstructural images, two RVEs were generated by the step-by-step packing method to reproduce a realistic 3D morphology of a cellular–dendritic structure composed of Al cells and a Si-rich network. Computational CPFEM analysis for the models at hand revealed a well-defined correlation between potential damage sites and anisotropy in ductility when the load was applied along and across the BD. The computational results suggested that the microscale strain gradients near the Al-Si interfaces would effectively delay damage initiation by reducing the number of potential damage sites (Figure 14).

**Figure 15:**
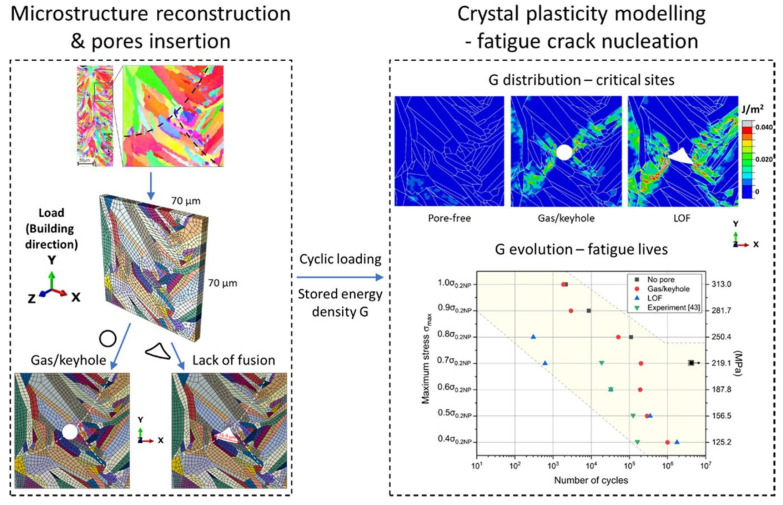
Quasi-3D structure-property modelling of as-built PBF-produced AlSi10Mg under cyclic loading. Three structure cases were considered, including pore free, gas/keyhole (a circular pore), and lack of fusion cases [198].

Going up to the grain scale, Cao et al. [198] utilised a quasi-3D microstructure-based CPFEM approach, which relies on the Orowan dislocation kinetics and a stored energy density criterion, to study numerically the effects of pore size and shape on the fatigue behaviour of LPBF AlSi10Mg. A 2D grain structure of a melt pool ‘triple junction’ region was reconstructed directly from an experimental orientation map and then extruded along the third direction to obtain a quasi-3D model. A single pore of circular or near-triangular shapes depicting a gas/keyhole defect and a lack of fusion defect, respectively, was placed in the melt pool overlap area — a region notoriously susceptible to pore formation during LPBF (Figure 15). The evolution of the stress–strain fields, dislocation density, and stored energy density in the vicinity of the pores was investigated in detail for each model microstructure. The authors observed local strain concentrations on the lateral sides of the pores under vertical tensile loading, which then started to propagate at an angle of 45° to the loading axis (Figure 15). This agrees well with the literature [199,200,201]. The CPFEM model was shown to capture basic features concerning the pore shape’s impact on the fatigue lifetime (Figure 15). According to [198], a lack of fusion pore leads to the reduction at high cyclic peak stress in orders of magnitude while both types of defects considered were shown to decrease predicted fatigue life. While the study [198] enables a better understanding of defect formation mechanisms, consideration of a larger computational domain would address the limitations of the approach employed in this work which incorporates a single triple junction region of melt pools, with none of the melt pools being considered in full, and a single pore. This, however, barely reflects the situation observed in AM samples which are produced with multiple tracks and in multiple layers.

**Figure 16:**
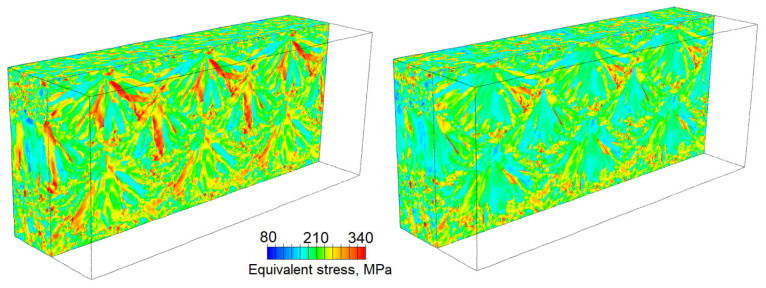
Equivalent stress fields in the middle sections of LPBF Al10SiMg RVEs at 5% tensile strain: the bulk of the melt pools is characterised by (**a**) randomly oriented and (**b**) cube-textured columnar grains [30].

**Figure 17:**
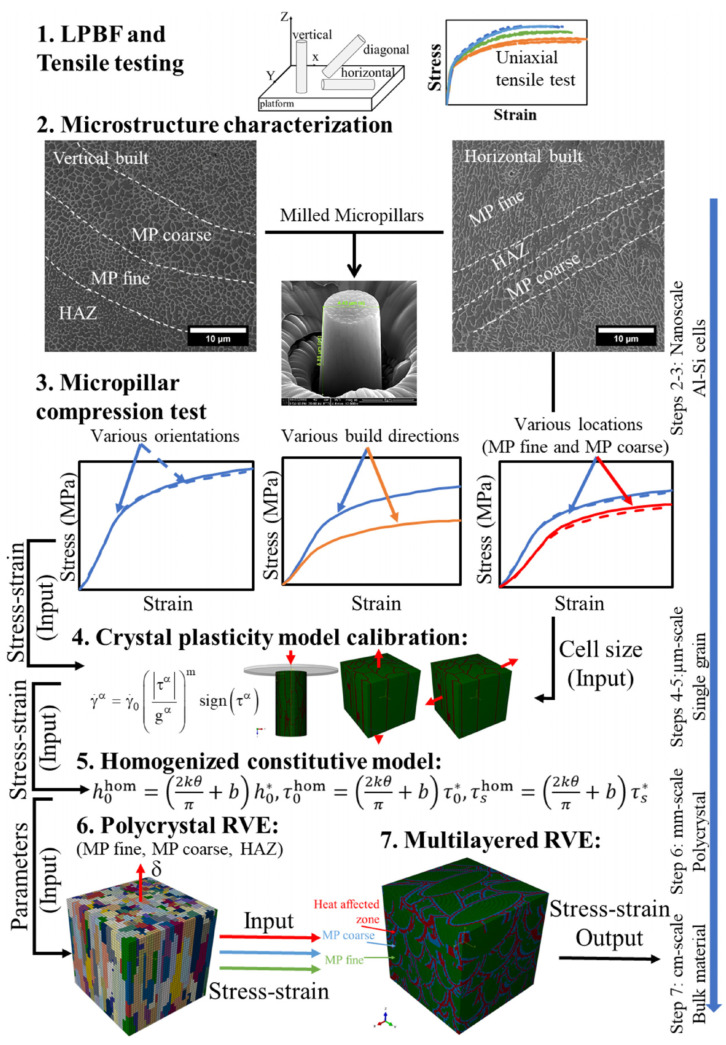
Multiscale methodology for predicting the impact of microstructure on the anisotropic mechanical properties of AlSi10Mg produced using LPBF [202].

Romanova et al. [29,30] employed an explicit CPFEM solver to study the grain-scale stress–strain fields developing in LPBF AlSi10Mg under tension. The explicit time integration procedure applicable to dynamic BVPs enabled a substantial reduction in computational costs as compared to implicit static simulations. A set of 3D synthetic models were generated by the step-by-step packing method [149] to reproduce a realistic grain morphology within multiple laser tracks and layers (Figure 16). Focusing on the analysis of local stress–strain values, the authors revealed a common tendency for the stresses to concentrate in the equiaxed grain regions along the melt pool boundaries. Comparison of the RVE models with different orientations of columnar grains showed that cube-textured grains in the central parts of the melt pools partially unloaded the adjacent equiaxed grain regions, thus reducing the portion of grains experiencing high local stresses (Figure 17).

Li et al. [202] developed a combined experimental/computational methodology to link the mechanical properties and microstructural features of an LPBF AlSi10Mg alloy in a bottom-up manner (Figure 17). The simulation procedure involved subsequent CPFEM analyses for the RVE models incorporating an Al-Si network at the dendritic scale, the grain morphology and orientations at the melt pool scale, and a fish-scale-like structure resolved experimentally with the naked eye. While each computational step was limited to consideration of the structural features and deformation events at a certain scale of consideration, the computational data obtained at a lower scale were subsequently used as input data on a larger scale. Using the computational/experimental methodology, the authors revealed that the origin of the anisotropic mechanical response typical of LPBF AlSi10Mg alloys was attributed to a dendritic structure with the cells elongated in the build direction. While the multiscale model was reported to overestimate the yield strength against the experimental data, it enables the prediction of optimal microstructural features tailoring superior mechanical properties of LPBF AlSi10Mg parts. Regrettably, the research [202] does not delve into the analysis of local stresses and strains.

The examples described above benchmark the advantages of and gaps in structure–property simulations for PBF AM. In particular, integrating synthetic microstructures simplifies the simulation process, reduces computational requirements, and enables faster simulations, which is useful for exploring a wide parameter space. The latter, however, would require extensive experimental support due to the necessity of statistics for generating synthetic microstructures. In many cases, synthetic microstructures might not accurately represent the complexities and variability of real PBF-produced microstructures, leading to potential inaccuracies in predicting material properties.

### 6.3. Process–Structure–Property–Performance Computational Analysis

Valuable as they are, the simulations treating synthetic microstructures fail to directly link AM processing with the material’s mechanical behaviour through PBF-induced microstructural features. Simulating the entire PSPP chain involves considering the complete AM process, from material deposition to cooling and solidification, resulting in more accurate and realistic representations of the final microstructure of a manufactured component. Furthermore, capturing the microstructure evolution during the process leads to a better understanding of how the process parameters affect the final material properties. As the studies integrating the PSPP computational analysis for PBF-produced materials are limited, we decided to consider both types of MAM, PBF and DED, in this section.

Researchers [32,108,201] were the first to implement the whole PSPP computational framework on the example of a multi-track and multi-layer PBF AM 316L print [32], a Ti-6Al-4V produced in a two-track two-layer PBF process [108], and DED two-track multi-layer printed 304L stainless steel [201]. Both independently proposed approaches [32,108] successively involved a solution of the thermal problem, the CA simulation of grain growth, calculations of grain-scale stress and strain fields, and a prediction of overall stress–strain curves. Each computational module generated input data for a subsequent step. The study [108] also considered the fatigue lifetime of an EPBF Ti-6Al-4V alloy.

**Figure 18:**
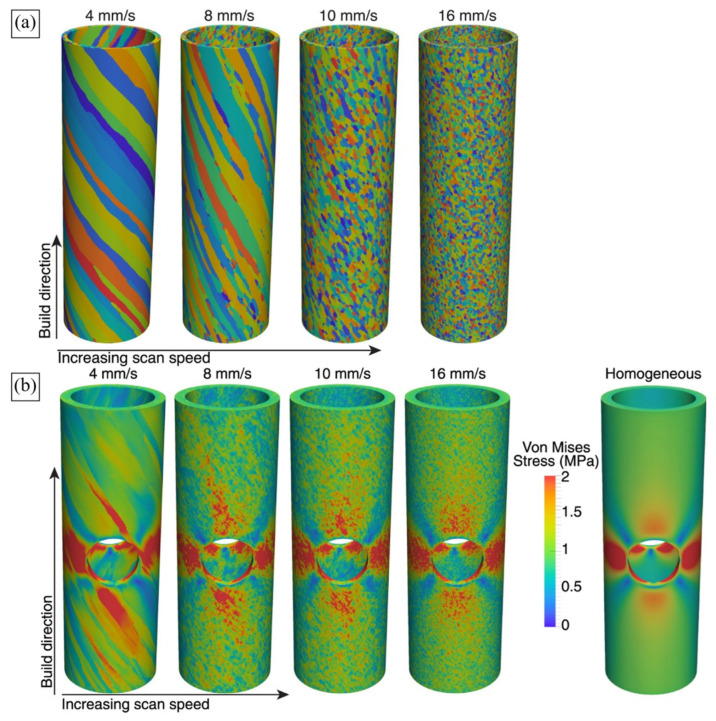
Results produced by the PSPP framework suggested in [201]: (**a**) model microstructures at the mid-surface of the tube’s wall thickness which are virtually printed at the different scan speeds and (**b**) von Mises stress distributions under torsional loading for grain structures shown in (**a**) and a homogeneous sample (on the right).

In contrast, the approach proposed in [201] did not include any thermal simulations of the process due to the particularities of the kMC approach that Rodgers and coworkers used for microstructure modelling. To replicate DED, they preset a moving melt pool and HAZ in terms of their geometry and mobility prefactor, which aimed to provide a ‘simulation surrogate to experimental conditions’ [140]. Apart from other PSPP computations disregarding a complex geometry of AM parts, Rodgers et al. [201] generated 3D DED-processed grain structures in thin-walled tubular-shaped AM parts with and without side holes (Figure 18). The AM process was simulated at four different scanning speeds to obtain microstructural models which benchmarked a gradual transition between columnar-grained samples and samples with equiaxed grains. Subsequent crystal elasticity analysis of local stress–strain fields and effective elastic properties was carried out to establish a link between the AM process, grain structure, elastic properties, and macroscale mechanical response to tensile and torsional loading. The authors concluded that the microstructure could induce local stress deviations from a homogeneous field within 50%, with the microstructural effects being more pronounced in the case of columnar grains. Interestingly, in the case of coarse columnar grains (the lowest scanning speed, 4 mm/s), the microstructure induced von Mises stress concentrations at the 45° angle to loading on top of the hole (Figure 18). Equiaxed grains structures (scanning speed of 10 and 16 mm/s) yielded local stress concentrations aligned with the loading direction, whereas the sample with finer columnar grains demonstrated transition between these behaviours.

Linking the computational modules, the authors [32,108,201] suggested powerful tools for the computer-aided optimisation of AM metals by varying the PBF processing parameters. While these efforts have contributed significantly to implementing the PSPP concept for MAM, it is important to acknowledge their inherent limitations. Among them are a quasi-3D formulation of the process-structure linkage and a plain-strain formulation of a BVP in [32] and the study of a two-track two-layer process in [108] which cannot be considered representative of a multi-track multi-layer PBF AM. The latter limitation led to performing the mechanical analysis for small grain subsets which were located within two tracks distributed one upon another. Unfortunately, spatial stress–strain distributions were overlooked in [108]. The studies [108,201] represent computational exercises which have not been validated experimentally, although they have briefly compared some of their numerical results against the literature in terms of the general trends or properties of conventionally manufactured materials. Furthermore, the scope of [201] was confined to the analysis of elastic deformation exclusively, thereby omitting any investigation into the plastic behaviour of DED-produced stainless steel.

**Figure 19:**
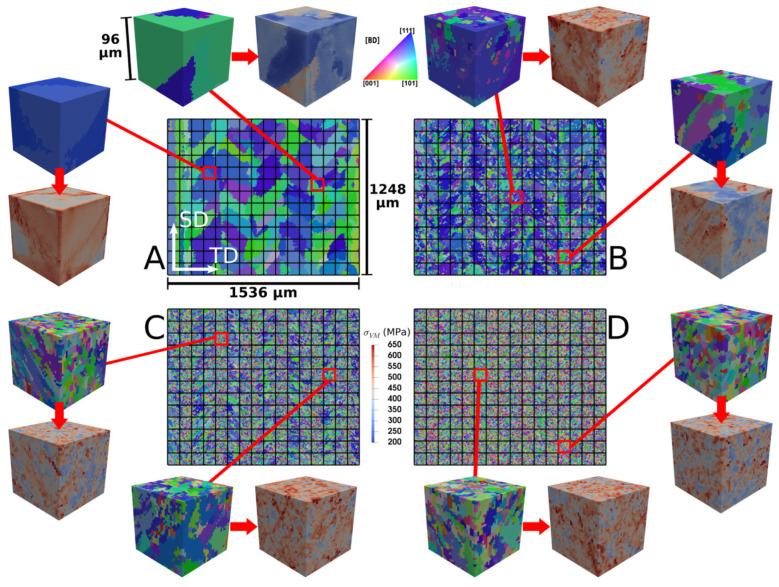
Results produced by the parallelised elasto-viscoplastic FFT framework for 4 model samples (**A**–**D**) of AM 316L steel which were virtually manufactured with different nucleation parameters and then subjected to uniaxial tension along the SD. The colours for the microstructures and stress distributions correspond to the legends for the inverse pole figure and von Mises stress, respectively [203].

A step forward along these lines was made in [203] that proclaimed ‘a need to leverage similar process–structure–property tools to predict the variability not only among different builds, but even within the same build.’ Accordingly, a computational framework was developed to simulate PSPP relationships of AM steel 316L produced by direct laser deposition. Grain solidification in a multi-pass, multi-layer DED process was predicted based on thermal history. The resulting grain structure was automatically segmented into smaller 3D RVEs whose mechanical response was calculated using CPFFT accounting for grain boundary strengthening (Figure 19). The effective mechanical properties of each RVE were automatically calculated and then used to generate a spatial property map for the built part. The study [203] has provided valuable insights for PSPP modelling framework implementation, though certain limitations should be acknowledged. Similarly to [201], the results were not validated with experimental data and thus at this stage might be considered as a pure modelling exercise. Single experimental data considered in the study are related to warm-rolled, traditionally manufactured SS316L steel [204]. Note that typically, melt pools created during DED are much larger than in PBF, and the grain structure is thus much coarser if no grain refinement is applied [205,206,207].

**Figure 20:**
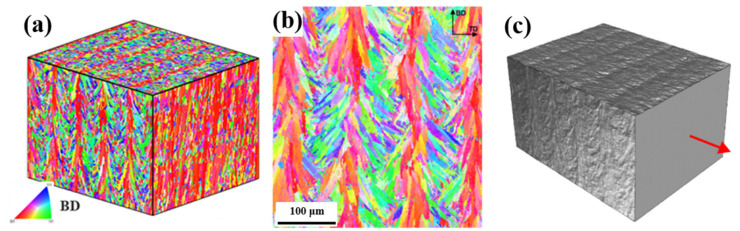
Output of the PSPP framework implemented in [109]: (**a**) model-predicted and (**b**) experimental grain structures of LPBF AlSi10Mg and corresponding deformation pattern obtained in high-fidelity CPFEM calculations.

**Figure 21:**
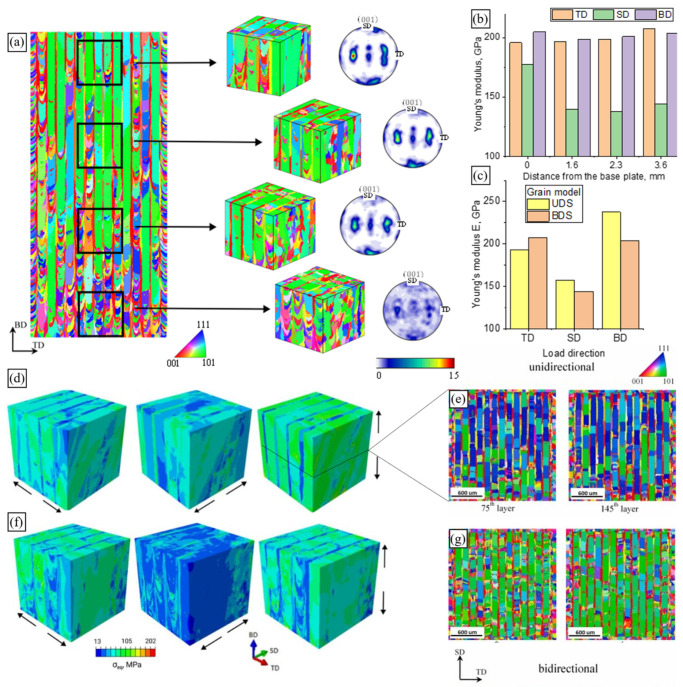
Results produced by the PSPP framework implemented in [166] for LPBF 316L steel: (**a**) TD-BD cross-section of a model-predicted grain structure and grain volume elements cut out from the reference microstructure model at the different distances from the base plate; (**b**) Young’s moduli of the models shown in (**a**); (**c**) Young’s moduli along the TD, SD, and BD calculated for LPBF 316L steel models which were printed using uni- and bidirectional scanning; (**d**,**f**) equivalent stress fields in LPBF grain structures printed by (**d**,**e**) unidirectional and (**f**,**g**) bidirectional scanning patterns under tension along TD, SD, and BD up to 0.035% strain and (**e**,**f**) respective grain structures in the SD-TD cross-sections. The black arrows on (**d**,**f**) show the loading direction.

In [17,109,166], the strategy of PSPP computational predictions for AM LPBF metals proposed by Zinovieva et al. [32] was extended to a 3D case of LPBF 316L steel and AlSi10Mg. The physically based CAFD simulations of grain nucleation and growth during LPBF enabled the authors to reproduce multi-track, multi-layer polycrystalline structures which well replicated EBSD data in terms of grain geometry and texture (Figure 20a and Figure 21a,e,g).

Subsequent high-fidelity FEM micromechanical simulations were performed for a set of smaller-sized grain sub-domains located in different regions of the reference CAFD models (Figure 21a). The effects of the grain structure and loading direction on the grain-scale stress–strain distributions in LPBF AlSi10Mg were addressed in [109]. Strongly inhomogeneous stress fields were found to develop at the grain scale where the stress tensor components and plastic strain localisation were shown to depend on the grain morphology and texture in a sophisticated way. Particularly, the grain-scale stress–strain fields demonstrated periodic layers of lower stress and higher plastic strains in the central parts of the tracks where the grains with <100>||BD orientations dominated [109]. Similarly, alternating through-the-thickness layers of lower and higher stresses were found to develop in LPBF 316L steel models [166] (Figure 21d,f). The local stress–strain fields and effective elastic properties depended on the loading direction and the grain features controlled, in turn, by the scan strategy and the position of the computational sub-domain with respect to the base plate (Figure 21a,b). Along with the numerical analysis, the effective elastic properties of the model microstructures were analytically estimated by the orientation distribution function-based Voigt–Reuss–Hill scheme to prove its applicability for predicting the elastic anisotropy of AM materials. Although Zinovieva et al. [166] considered the scanning pattern’s impact on the very beginning of plastic deformation in LPBF steel, it is worth keeping in mind that the study was carried out under elastic loading, similar to [201].

## 7. Summary and Future Directions

Metal AM produces parts with intricate hierarchical microstructures and mechanical properties which are very different from conventionally produced parts. Synergy between experiments and modelling is crucial to enhance the understanding of the mechanical behaviour of AM materials and to support the qualification and certification of AM parts.

This review critically analysed computational approaches which target a deeper grasp of the mechanics of AM materials and offer a means to accelerate the exploration of a vast design space. In addition, this review discussed the foundational aspects which should be considered in the mechanical modelling of AM materials. Among them are microstructural features inherent to AM materials at different spatial scales (Section 2) and the advanced modelling approaches employed to describe PBF-produced microstructures (Section 4). Section 5 focused on the BVP formulation tailored for microstructure-based mechanical simulations of AM alloys, including kinematics, constitutive laws, hardening mechanisms, and numerical implementations. Central to our discussion is the PSPP concept which should leverage high-performance computing modelling capabilities to support the community in the exploration of AM’s adaptability and potential for tailored mechanical properties.

PBF AM materials exhibit a microstructure hierarchy profoundly influenced by process parameters. The melt pool pattern, grains (µm-mm), cellular–dendritic sub-grain structures (µm), and precipitates (nm) each affect the mechanical properties. Melt pool boundaries can act as nucleation sites for new grains and/or cracks. Grains in AM materials are typically columnar and have a preferred orientation, which induces the anisotropy of mechanical properties. A very fine cellular–dendritic substructure, characteristic of PBF, leads to the superior yield stress, hardness, and/or UTS of AM alloys.

The ability to characterise, predict, and manipulate the microstructural evolution during the AM process is central to unlocking enhanced material performance—this is where the experiment-modelling synergy is of key importance. Currently the literature tends to consider the models of different scales separately. Given the complexity of the metal AM process, multiscale models should be employed in the future. Microstructure models can be classified into physically based—considering thermodynamics, kinetics, and other fundamental principles to simulate microstructural evolution—and geometrically based—generating microstructures, which are statistically similar to those observed experimentally, without capturing the dynamics of their formation. In contrast to geometrically based models, physically based ones offer insights into process and property optimisation but require knowledge of material properties, accurate boundary and initial conditions and often necessitate high computational power.

The next step after creating a digital twin of the material’s microstructure is to define a mechanical BVP along with its numerical implementation. Constitutive laws for the micromechanical modelling of PBF AM range from those disregarding crystal lattice effects in the simplest case to intricate physically based crystal plasticity models. This paper summarises hardening laws applied in the micromechanical modelling of AM metals.

Pioneering research on the structure–property modelling of AM alloys has been performed in a 2D setting and enabled the rapid computational analysis of their mechanical behaviour due to reduced spatial complexity. Valuable as they are, 2D simulations miss the 3D nature of the material behaviour, which can be resolved by 3D mechanical simulations using synthetic microstructures or the more holistic PSPP computational analysis. While the first approach is limited to evaluating the microstructural effects on mechanical properties, the PSPP analysis broadens the scope to encompass the entire lifecycle, from processing to end-use. Three-dimensional microstructure-informed mechanical simulations can provide stress–strain curves, failure modes, or other mechanical property data. The PSPP analysis offers a chain of results and can help in optimising process parameters for desired outcomes or predicting the lifespan and durability of manufactured parts, with it being more computationally demanding, however, due to the integration of multiple stages.

This critical analysis has identified several gaps in the current AM modelling landscape. Among them are scarce PSPP simulations, particularly pertaining to PBF AM, and limited attention to local stresses and strains which should support the accurate capture and deeper understanding of the intricacies of material behaviour. While modelling approaches towards microstructure-informed mechanical simulations have progressed significantly, there is still a lack of experimental validation for existing and newly developed models, which restrains their practical adoption. An ideal trajectory for future AM research would involve a concerted effort to create full experimental datasets that would help validate modelling outcomes. Combining experimental and modelling efforts in collaborative projects can help in obtaining and validating data across scales and in solidifying our understanding of the intricate PSPP relationships. This enriched understanding, in turn, could serve as a foundation for facilitating the design of novel materials specifically tailored for AM. Expanding the horizons of PSPP simulations for AM materials to embrace diverse loading conditions, such as biaxial loading, fatigue, and impact, is an avenue ripe for exploration. Investigating the microstructure effects on process-induced residual stresses presents an intriguing dimension that is still relatively uncharted. Currently being limited to several commercial alloys traditionally used in PBF AM, the scope of modelling efforts should broaden to encompass diverse materials, including those enabled by the technology, such as functionally graded materials, bio-inspired materials, and metamaterials.

The advances along these lines will not only bridge current gaps but also have a significant impact on the design, qualification, and certification of AM metal parts. The potential to propel these methodologies from research insights to industry standards lies in addressing the identified gaps. We believe that the methodologies summarised in this review lay the groundwork for further innovation.

## Figures and Tables

**Table 1:** Overview of hardening laws considered in the micromechanical simulations of PBF materials.

Topic, Reference	Highlights	Hardening Law
Damage modelling of alloys produced by LPBF [146]	Two-dimensional FE structure–property model developed for uniaxial tensile loading of as-built LPBF 316L steel. Incorporates a simplistic synthetic grain structure that fits a melt pool (Figure 7a). Implemented in the FE commercial software Abaqus using a user material (UMAT) subroutine. Compared with an experimental stress–strain curve of the as-built LPBF 316L steel.	${\dot{\tau }}_{c}^{\left(\alpha \right)}=\sum_{\beta }h_{\alpha \beta }{\dot{\gamma }}^{\beta }$. (20) Here, $h_{\alpha \beta }$ areare the hardening moduli, including $h_{\alpha \alpha }$ (no sum on α) and $h_{\alpha \beta }$ ( (α≠β) which denote self and latent hardening moduli, respectively, as introduced by Peirce et al. [178], $h_{\alpha \alpha }=h\left(\gamma \right)=h_{0}\sec h^{2}\left|\frac{h_{0}\gamma }{\tau_{s}-\tau_{0}}\right|$, $h_{\alpha \beta }=qh\left(\gamma \right)$. Here, $h_{0}$ is the initial hardening modulus; γ stands for accumulated shear strain; $\tau_{s}$ is the saturation stress; $\tau_{0}$ is the initial stress and q denotes the ratio of latent to self-hardening. Latent hardening $h_{\alpha \beta }$ represents the increment of flow stress on the slip system α due to a shear increment on the slip system β. As pointed out by Peirce et al. [178], the components of the hardening matrix represent ‘the most elusive parameters’ in the constitutive equations. In a later study [179], Taheri Andani and coworkers adapted the model proposed in [180] where they considered the slip system hardening model as $h_{\alpha \beta }=\left[q+\left(1-q\right)\delta^{\alpha \beta }\right]h^{\beta }$ (no sum on β), where a single slip hardening rate $h^{\beta }=h_{0}{\left(1-\frac{s^{\beta }}{s_{s}}\right)}^{a}$; $\delta^{\alpha \beta }$ is the Kronecker delta; and $h_{0}$; a, $s_{s}$ are the slip system hardening parameters which were set to be identical for all slip systems.
Micromechanical modelling of single track deformation, phase transformation and residual stress evolution during laser surface remelting [175]	Two-dimensional FE structure–property model developed to estimate residual stresses that are formed due to the laser surface remelting of H13 tool steel. Incorporates a simplistic synthetic equiaxed grain structure and takes into account martensitic and austenitic phases. Implemented in the FE software Zset [181]. When determining single crystal model parameters, the authors compared a calculated macroscopic stress–strain curve with an experimental one for H13 samples that were cast and heat-treated [182].	Martensite: $\tau_{c}^{\left(\alpha \right)}=\sqrt{{\left(\tau_{self}^{\left(\alpha \right)}\right)}^{2}+{\left(\tau_{LT}^{\left(\alpha \right)}\right)}^{2}}+\tau_{HP}+\tau_{0/SS}^{\left(\alpha \right)}$. (21) Here, the self-hardening resistance $\tau_{self}^{\left(\alpha \right)}=\mu b^{m}\sqrt{\alpha_{self}\rho^{\left(\alpha \right)}}$$,\;\mathrm{the}\;\mathrm{line}\;\mathrm{tension}\;\mathrm{resistance}\;\tau_{LT}^{\left(\alpha \right)}=\max\left[\frac{\alpha^{\left(\alpha \right)}\mu b^{m}}{\lambda^{\left(\alpha \right)}-l_{c}}-\tau_{eff},0\right]$$,\;\mathrm{the}\;\mathrm{Hall}–\mathrm{Petch}\;\mathrm{term}\;\tau_{HP}=\frac{\mu }{\mu \left(300K\right)}\frac{K_{HP}}{\sqrt{d_{bcc}}}$$,\;\mathrm{and}\;\mathrm{the}\;\mathrm{solid}\;\mathrm{solution}\;\mathrm{strengthening}\;\tau_{0/SS}^{\left(\alpha \right)}$ which is usually defined as a constant, are considered. Here,μ$\;\mathrm{and}\;\mu \left(300K\right)$$\;\mathrm{is}\;\mathrm{the}\;\mathrm{shear}\;\mathrm{modulus}\;\mathrm{at}\;\mathrm{the}\;\mathrm{current}\;\mathrm{and}\;\mathrm{reference}\;\mathrm{temperatures},\;\mathrm{respectively};\;b^{m}$$\;\mathrm{stands}\;\mathrm{for}\;\mathrm{the}\;\mathrm{length}\;\mathrm{of}\;\mathrm{the}\;\mathrm{Burgers}\;\mathrm{vector};\;\alpha_{self}$ is the hardening coefficient characterising the dislocation network interactions [183]; $\rho^{\left(\alpha \right)}$ represents the dislocation density on the slip system α$;\;\alpha^{\left(\alpha \right)}$$\;\mathrm{denotes}\;\mathrm{the}\;\mathrm{mean}\;\mathrm{obstacle}\;\mathrm{strength};\;\lambda^{\left(\alpha \right)}$$\;\mathrm{is}\;\mathrm{the}\;\mathrm{average}\;\mathrm{obstacle}\;\mathrm{spacing};\;\mathrm{and}\;l_{c}$ stands for the minimum length of the screw segment. $\tau_{eff}=\left\{\begin{array}{c} \left|\tau^{\left(\alpha \right)}\right|-\tau_{c}^{\left(\alpha \right)},\;\;\;if\;\left|\tau^{\left(\alpha \right)}\right|>\tau_{c}^{\left(\alpha \right)}\\ 0,\;\;\;otherwise \end{array}\right.$ is the effective stress, i.e., mean stress driving dislocation motion; $K_{HP}$$\;\mathrm{represents}\;\mathrm{the}\;\mathrm{Hall}–\mathrm{Petch}\;\mathrm{constant};\;\mathrm{and}\;d_{bcc}$$\;\mathrm{stands}\;\mathrm{for}\;\mathrm{the}\;\mathrm{effective}\;\mathrm{grain}\;\mathrm{size}.\;\mathrm{At}\;\mathrm{elevated}\;\mathrm{temperatures}\;\mathrm{and}\;\mathrm{as}\;\mathrm{the}\;\mathrm{average}\;\mathrm{obstacle}\;\mathrm{spacing}\;\lambda^{\left(\alpha \right)}$$\;\mathrm{approaches}\;1/\sqrt{\rho_{obs}}$ the line tension model goes to the classical formulation proposed by Taylor. The average obstacle strength is a function of the densities of different defects, $\alpha^{\left(\alpha \right)}=\frac{1}{\sqrt{\rho_{obs}^{\left(\alpha \right)}}}\sqrt{\sum_{\alpha \neq \beta }a_{eff}^{\alpha \beta }\rho_{dis}^{\beta }+\rho_{carb}+\rho_{SC}}$$.\;\mathrm{The}\;\mathrm{interaction}\;\mathrm{matrix}\;\mathrm{between}\;\mathrm{dislocation}\;\mathrm{slip}\;\mathrm{systems}\;\mathrm{is}\;\mathrm{defined}\;\mathrm{as}\;a_{eff}^{\alpha \beta }={\left(0.2+0.8\frac{\ln\left(0.35b^{\left(\alpha \right)}\sqrt{\rho_{obs}}\right)}{\ln\left(0.35b^{\left(\alpha \right)}\sqrt{\rho_{ref}}\right)}\right)}^{2}a^{\alpha \beta }$$,\;\mathrm{where}\;a^{\alpha \beta }$$\;\mathrm{denotes}\;\mathrm{the}\;\mathrm{interaction}\;\mathrm{constants};\;\rho_{ref}$$\;\mathrm{represents}\;\mathrm{the}\;\mathrm{reference}\;\mathrm{total}\;\mathrm{dislocation}\;\mathrm{density}\;\mathrm{wherein}\;\mathrm{the}\;\mathrm{interaction}\;\mathrm{constants}\;\mathrm{are}\;\mathrm{found},\;\mathrm{for}\;\mathrm{example},\;\mathrm{with}\;\mathrm{dislocation}\;\mathrm{dynamics}\;\mathrm{simulations};\;\rho_{obs}$$\;\mathrm{is}\;\mathrm{the}\;\mathrm{average}\;\mathrm{obstacle}\;\mathrm{density}\;\mathrm{defined}\;\mathrm{as}\;\mathrm{the}\;\mathrm{planar}\;\mathrm{defect}\;\mathrm{density}\;\rho_{obs}^{\alpha }=\sum_{\alpha \neq \beta }\rho_{dis}^{\beta }+\rho_{carb}+\rho_{SC}$$;\;\rho_{dis}^{\beta }$ represents the dislocation density on slip system β$;\;\mathrm{and}\;\rho_{carb}$$\;\mathrm{and}\;\rho_{SC}$ are the planar densities of carbides and solute clusters, respectively. A similar hardening model was adapted for austenite: $\tau_{c}^{\left(\gamma \right)}=\tau_{eff/SS}^{\left(\gamma \right)}+\mu^{a}b^{a}\sqrt{\sum_{a=1}^{12}a_{a-eff}^{\alpha \beta }\rho^{\beta }+a_{carb}\rho_{carb}^{a}+a_{SC}\rho_{SC}^{a}}+\tau_{HP}^{a}$. (22) Here, $\tau_{eff/SS}^{\left(\gamma \right)}=\frac{\mu^{a}}{\mu \left(300K\right)}\tau_{0/SS}^{\left(\gamma \right)}$$\;\mathrm{represents}\;\mathrm{the}\;\mathrm{solid}\;\mathrm{solution}\;\mathrm{strengthening}\;\mathrm{at}\;\mathrm{room}\;\mathrm{temperature}\;\mathrm{and}\;\tau_{HP}^{a}$ is the Hall–Petch term. Dislocation density evolution is estimated using a classical equation which describes the evolving mean free path during the accumulation of dislocations. The model is largely adopted from [184].
Microstructural effects on the elasto-viscoplastic deformation of dual-phase Ti-6Al-4V produced by PBF [185]	Three-dimensional fast Fourier transform (FFT) structure–property model developed for the uniaxial tensile loading of as-built PBF Ti-6Al-4V. Incorporates a synthetic grain structure that accounts for prior β grains (both elongated and equiaxed) and α laths. Equation (23) is calibrated with the experimental stress–strain curve of the as-built EPBF Ti-6Al-4V [186].	A modified version of the Voce hardening model $\tau_{c}^{\left(\alpha \right)}=\tau_{0}^{\left(\alpha \right)}+\left(\tau_{1}^{\left(\alpha \right)}+\theta_{1}^{\left(\alpha \right)}\Gamma \right)\left(1-\exp\left(-\Gamma \left|\frac{\theta_{0}^{\left(\alpha \right)}}{\tau_{1}^{\left(\alpha \right)}}\right|\right)\right)$, (23) $\;\mathrm{with}\;\mathrm{four}\;\mathrm{parameters}\;\mathrm{for}\;\mathrm{each}\;\mathrm{slip}\;\mathrm{system}.\;\mathrm{Here},\;\tau_{0}^{\left(\alpha \right)}$$\;\mathrm{represents}\;\mathrm{the}\;\mathrm{initial}\;\mathrm{CRSS};\;\tau_{1}^{\left(\alpha \right)}$$\;\mathrm{denotes}\;\mathrm{the}\;\mathrm{initial}\;\mathrm{hardening}\;\mathrm{rate};\;\theta_{0}^{\left(\alpha \right)}$$\;\mathrm{is}\;\mathrm{the}\;\mathrm{asymptotic}\;\mathrm{hardening}\;\mathrm{rate};\;\mathrm{and}\;\theta_{1}^{\left(\alpha \right)}$ stands for the back-extrapolated CRSS.
CP modelling of the anisotropic tensile behaviour of LPBF Ti-6Al-4V [36]	Three-dimensional FFT structure–property model developed for the uniaxial tensile loading of the as-built LPBF Ti-6Al-4V in three different directions. Incorporates a synthetic grain structure that accounts for elongated prior β grains and α laths. Implemented in the FFT software DAMASK [157]. Equation (24) is calibrated with experimental the stress–strain curves of the heat-treated LPBF Ti-6Al-4V ELI.	The slip resistance ${\dot{\tau }}^{\left(\alpha \right)}=h_{0}\left(1+h_{int}^{\left(\alpha \right)}\right)\sum_{\beta =1}^{N_{s}}\left|{\dot{\gamma }}^{\beta }\right|{\left|1-\frac{{\dot{\tau }}^{\beta }}{{\dot{\tau }}_{\infty }^{\beta }}\right|}^{a-1}\left(1-\frac{{\dot{\tau }}^{\beta }}{{\dot{\tau }}_{\infty }^{\beta }}\right)h^{\alpha \beta }$, (24) $\mathrm{where}\;h_{0}$$\;\mathrm{stands}\;\mathrm{for}\;\mathrm{an}\;\mathrm{overall}\;\mathrm{hardening}\;\mathrm{parameter}\;\mathrm{of}\;\mathrm{unit}\;\mathrm{stress};\;h_{int}^{\left(\alpha \right)}$$\;\mathrm{is}\;\mathrm{the}\;\mathrm{slip}\;\mathrm{system}’s\;\mathrm{specific}\;\mathrm{corrections}\;(\mathrm{dimensionless})\;\mathrm{to}\;h_{0}$$;\;h^{\alpha \beta }$$\;(\mathrm{dimensionless})\;\mathrm{describes}\;\mathrm{the}\;\mathrm{interactions}\;\mathrm{between}\;\mathrm{different}\;\mathrm{slip}\;\mathrm{systems};\;h^{\alpha \alpha }=1$$\;\mathrm{is}\;\mathrm{used}\;\mathrm{for}\;\mathrm{the}\;\mathrm{interaction}\;\mathrm{of}\;a\;\mathrm{slip}\;\mathrm{system}\;\mathrm{with}\;\mathrm{itself};\;\mathrm{and}\;{\dot{\tau }}_{\infty }^{\beta }$ denotes the saturation value of the slip resistance.
CP modelling of the structure–property relationship in LPBF Ti-6A-4V [152]	Three-dimensional FE structure–property model developed for the uniaxial tensile, compressive, and cyclic loading of the as-built LPBF Ti-6Al-4V. Incorporates a synthetic grain structure that accounts for elongated prior β grains and α’ laths. Implemented in Abaqus using a UMAT subroutine. The results are compared with the experimental stress–strain curves obtained under tension, compression, and cyclic loading along the BD of LPBF Ti-6Al-4V.	$\mathrm{The}\;\mathrm{shear}\;\mathrm{slip}\;\mathrm{rate}\;\mathrm{follows}\;a\;\mathrm{Norton}-\mathrm{type}\;\mathrm{law},\;\mathrm{similar}\;\mathrm{to}\;\mathrm{Equation}\;(15),\;\mathrm{and}\;\mathrm{is}\;a\;\mathrm{function}\;\mathrm{of}\;\mathrm{the}\;\mathrm{resolved}\;\mathrm{shear}\;\mathrm{stress}\;\tau^{\left(\alpha \right)}$$,\;\mathrm{back}\;\mathrm{stress}\;\chi^{\left(\alpha \right)}$$,\;\mathrm{threshold}\;\mathrm{stress}\;\kappa^{\left(\alpha \right)}$$;\;\mathrm{and}\;\mathrm{the}\;\mathrm{drag}\;\mathrm{stress}\;D_{f}^{\left(\alpha \right)}$, where $,\left.\begin{array}{c} {\dot{\chi }}^{\left(\alpha \right)}=h{\dot{\gamma }}^{\left(\alpha \right)}-h_{D}\chi^{\left(\alpha \right)}\left|{\dot{\gamma }}^{\left(\alpha \right)}\right|\\ \kappa^{\left(\alpha \right)}=\frac{\kappa_{y}}{\sqrt{d^{\left(\alpha \right)}}}+\kappa_{s}^{\left(\alpha \right)}\\ \begin{array}{c} {\dot{\kappa }}_{s}^{\left(\alpha \right)}=-\phi \kappa_{s}^{\left(\alpha \right)}\left|{\dot{\gamma }}^{\left(\alpha \right)}\right|\\ D_{f}^{\left(\alpha \right)}=\tau_{CRSS}^{\left(\alpha \right)}-\kappa_{s}^{\left(\alpha \right)} \end{array} \end{array}\right\}$ (25) Here, h$,\;h_{D}$ and ϕ$\;\mathrm{are}\;\mathrm{constant}\;\mathrm{parameters};\;\kappa_{y}$$\;\mathrm{denotes}\;\mathrm{the}\;\mathrm{Hall}–\mathrm{Petch}\;\mathrm{slope};\;d^{\left(\alpha \right)}$ refers to the microstructural dimension which relates to the free slip length on the slip system α$;\;\mathrm{and}\;\tau_{CRSS}^{\left(\alpha \right)}$ stands for CRSS.
Macroscale and microscale stress–strain relations of LPBF AlSi10Mg alloy [187]	Three-dimensional FFT structure–property model developed for the uniaxial tensile loading of as-built LPBF AlSi10Mg. Incorporates a synthetic grain structure (columnar or equiaxed), Si particles, and the porosity with a prescribed volume fraction. Implemented in DAMASK [157]. Compared the computationally obtained stress–strain curves with the experimental one from the in situ synchrotron X-ray diffraction experiment [188].	${\dot{\tau }}_{c}^{\left(\alpha \right)}=\sum_{\beta }h_{0}q_{uv}{\left(1-\tau_{c}^{\left(\beta \right)}/\tau_{s}\right)}^{a}\left|{\dot{\gamma }}^{\beta }\right|$$,\;{\left.\tau_{c}^{\left(\beta \right)}\right|}_{t=0}=\tau_{0}$ (26) $\mathrm{Here},\;h_{0}$, a$,\;\tau_{s}$$\;\mathrm{are}\;\mathrm{the}\;\mathrm{slip}\;\mathrm{hardening}\;\mathrm{parameters}\;\mathrm{and}\;\tau_{0}$ stands for the initial slip resistance.
Phase stress partition and its correlation with the mechanical anisotropy of LPBF AlSi10Mg [189]	Three-dimensional FE structure–property models developed for uniaxial tensile loading of the as-built LPBF AlSi10Mg in two different directions. Incorporate a synthetic Al-Si cellular substructure. Implemented in Abaqus using a UMAT subroutine. The results are compared with the experimental stress–strain curves obtained under tension along the BD and TD of LPBF AlSi10Mg [190].	The traditional CP model 191 and the mechanism-based strain gradient crystal plasticity model (MSG-CP) [192] were employed. The former considered the hardening law suggested by Peirce et al. [178], as described in Equation (20). The MSG-CP defined the effective slip resistance following the Taylor hardening relation: $\tau_{c\_T}^{\left(\alpha \right)}=\tau_{c0}\sqrt{{\left(\tau_{c}^{\left(\alpha \right)}/\tau_{c0}\right)}^{2}+l\eta_{G}^{\left(\alpha \right)}}$, (27) $\;\mathrm{Here},\;\tau_{c0}$$\;\mathrm{is}\;\mathrm{the}\;\mathrm{reference}\;\mathrm{slip}\;\mathrm{resistance}\;(\mu /\tau_{c0}\approx 100$), $\eta_{G}^{\left(\alpha \right)}$$\;\mathrm{represents}\;\mathrm{the}\;\mathrm{effective}\;\mathrm{strain}\;\mathrm{gradient},\;l=\left(a^{2}\mu^{2}b\right)/\tau_{c0}^{2}$$\;\mathrm{stands}\;\mathrm{for}\;\mathrm{the}\;\mathrm{intrinsic}\;\mathrm{material}\;\mathrm{length},\;a\in \left[0.2;0.5\right]$ is the empirical coefficient, and μ$\;\mathrm{stands}\;\mathrm{for}\;\mathrm{the}\;\mathrm{shear}\;\mathrm{modulus}.\;\mathrm{The}\;\mathrm{MSG}-\mathrm{CP}\;\mathrm{considers}\;\tau_{c\_T}^{\left(\alpha \right)}$$\;\mathrm{defined}\;\mathrm{by}\;\mathrm{Equation}\;(27)\;\mathrm{instead}\;\mathrm{of}\;\tau_{c}^{\left(\alpha \right)}$ in Equation (13).

## Data Availability

As this is a review paper, no new data were created.
